# Identifying urban hotspots of dengue, chikungunya, and Zika transmission in Mexico to support risk stratification efforts: a spatial analysis

**DOI:** 10.1016/S2542-5196(21)00030-9

**Published:** 2021-05-05

**Authors:** Felipe Dzul-Manzanilla, Fabián Correa-Morales, Azael Che-Mendoza, Jorge Palacio-Vargas, Gustavo Sánchez-Tejeda, Jesus F González-Roldan, Hugo López-Gatell, Adriana E Flores-Suárez, Hector Gómez-Dantes, Giovanini E Coelho, Haroldo S da Silva Bezerra, Norma Pavia-Ruz, Audrey Lenhart, Pablo Manrique-Saide, Gonzalo M Vazquez-Prokopec

**Affiliations:** a Centro Nacional de Programas Preventivos y Control de Enfermedades (CENAPRECE), Ministry of Health, Mexico; b Collaborative Unit for Entomological Bioassays (UCBE), Campus de Ciencias Biologicas y Agropecuarias, Universidad Autonoma de Yucatan, Merida, Yucatan, Mexico; c Centro de Investigaciones Regionales Hideyo Noguchi, Universidad Autonoma de Yucatan, Merida, Yucatan, Mexico; d Servicios de Salud de Yucatan, Merida, Yucatan; e Sociedad Mexicana de Salud Publica, Mexico City, Mexico; f Subsecretaria de Prevencion y Promocion de la Salud, Mexico City, Mexico; g Facultad de Ciencias Biologicas Universidad Autónoma de Nuevo Leon, San Nicolas de los Garza, Nuevo Leon, Mexico; h Health Systems Research Center, National Institute of Public Health, Cuernavaca, Mexico; i Pan American Health Organization (PAHO), Department of Communicable Diseases and Environmental Determinants of Health, Washington DC, USA; j Centers for Disease Control and Prevention, Atlanta, GA, USA; k Department of Environmental Sciences, Emory University, Atlanta, GA, USA

## Abstract

**Background:**

Effective *Aedes aegypti* control is limited, in part, by the difficulty in achieving sufficient intervention coverage. To maximise the effect of vector control, areas with persistently high numbers of *Aedes*-borne disease cases could be identified and prioritised for preventive interventions. We aimed to identify persistent *Aedes*-borne disease hotspots in cities across southern Mexico.

**Methods:**

In this spatial analysis, geocoded cases of dengue, chikungunya, and Zika from nine endemic Mexican cities were aggregated at the census-tract level. We included cities that were located in southern Mexico (the arbovirus endemic region of Mexico), with a high burden of dengue cases (ie, more than 5000 cases reported during a 10-year period), and listed as high priority for the Mexican dengue control and prevention programme. The Getis-Ord Gi*(d) statistic was applied to yearly slices of the dataset to identify spatial hotspots of each disease in each city. We used Kendall's W coefficient to quantify the agreement in the distribution of each virus.

**Findings:**

128 507 dengue, 4752 chikungunya and 25 755 Zika clinical cases were reported between Jan 1, 2008, and Dec 31, 2016. All cities showed evidence of transmission heterogeneity, with a mean of 17·6% (SD 4·7) of their total area identified as persistent disease hotspots. Hotspots accounted for 25·6% (SD 9·7; range 12·8–43·0) of the population and 32·1% (10·5; 19·6–50·5) of all *Aedes*-borne disease cases reported. We found an overlap between hotspots of 61·7% for dengue and Zika and 53·3% for dengue and chikungunya. Dengue hotspots in 2008–16 were significantly associated with dengue hotspots detected during 2017–20 in five of the nine cities. Heads of vector control confirmed hotspot areas as problem zones for arbovirus transmission.

**Interpretation:**

This study provides evidence of the overlap of *Aedes*-borne diseases within geographical hotspots and a methodological framework for the stratification of arbovirus transmission risk within urban areas, which can guide the implementation of surveillance and vector control.

**Funding:**

USAID, the US Centers for Disease Control and Prevention, the Canadian Institutes of Health Research, International Development Research Centre, Fondo Mixto CONACyT (Mexico)-Gobierno del Estado de Yucatan, and the US National Institutes of Health.

**Translation:**

For the Spanish translation of the abstract see Supplementary Materials section.

## Introduction

The arboviral diseases dengue, chikungunya, and Zika, transmitted primarily through the bites of female *Aedes aegypti*, are substantial global public health problems in most of the tropics. Dengue is the most problematic because of its widespread distribution and the recurrence of large-scale outbreaks that exceed the capabilities of most public health systems. Dengue is present in 128 countries, and 3·97 billion people are estimated to be at risk of infection,^1^ with projected estimates showing an increasing trend in illness and fatalities.^2^ The Americas and Asia are the regions most affected by dengue^3^, ^4^ (and more recently by chikungunya^5^ and Zika^6^). Because the only commercially licensed dengue vaccine has low efficacy and no prophylactic or therapeutic medications exist for dengue, chikungunya, or Zika, vector control is the principal method for curtailing transmission and containing outbreaks.^7^

In Mexico, dengue transmission occurs in 29 out of 32 states.^8^ Estimates have shown that annually the country has between 75 203 and 355 343 dengue cases, incurring an economic cost of US$149 million to $257 million per year.^9^, ^10^ All four dengue virus serotypes circulate in Mexico and, since the introduction of chikungunya in 2014^11^ and Zika in 2015,^12^ Mexico's public health system has been exploring integrated vector control strategies to confront these arboviral diseases using risk stratification and the identification of high-risk areas.^8^ Almost 80% of all dengue cases are in the southeast region of Veracruz, Yucatan, Morelos, Guerrero, Chiapas, Oaxaca, and Tabasco. During 2020, a 50% decrease in the number of cases was registered and the southern states only contributed to 14% of confirmed cases.

**Research in context**

**Evidence before this study**

For decades, dengue control programmes have based their strategies on universal and reactive insecticide-based vector control actions, which have failed to contain outbreaks or the spread of emerging *Aedes*-borne diseases such as chikungunya or Zika. A renewed perspective, supported by theoretical evidence, focuses on the identification of areas that concentrate a large fraction of *Aedes*-borne disease cases as an approach for reframing vector control actions. We searched our personal libraries for papers relevant to the transmission and control of dengue in urban environments, including the landmark review by Achee and colleagues in 2015. A study in the metropolitan area of Merida, Yucatán, Mexico, applied spatial statistics to passive surveillance data and found that approximately 42% of all *Aedes*-borne disease cases were associated with persistent geographical transmission hotspots. The generalisability of such findings to other localities in Mexico remains to be assessed.

**Added value of this study**

This study extends and reports new evidence of heterogeneity and overlap in the transmission of *Aedes*-borne viruses in nine cities in Mexico. Dengue transmission hotspots show temporal consistency and account for 32% of all reported cases. Furthermore, dengue hotspots overlapped with chikungunya and Zika occurrences and, in most cities, were the first areas within cities to report the presence of these emerging diseases.

**Implications of all the available evidence**

Our findings show temporal and geographical patterns of *Aedes*-borne diseases in cities across southern Mexico, supporting the importance of hotspot detection to better inform vector control interventions. Future work will investigate the efficacy of preventive interventions focused on hotspots as a new paradigm for the prevention of *Aedes*-borne diseases. This approach represents a shift away from blanket control strategies towards a more focused and rational management of *Aedes* in urban areas that is based on risk stratification.

A further refinement of integrated vector management proposed in Mexico involves the analysis of historical dengue, chikungunya, and Zika data to identify areas within cities in which individuals with these diseases concentrate (hotspots). This form of risk stratification^13^ was assessed in the city of Merida, Yucatan, where spatial analyses identified that 42% of dengue cases were found in 27% of the city, and that these hotspot areas were also the introduction points of Zika and chikungunya.^14^ The Merida findings prompted interest in further validating this methodology of risk stratification in cities of differing size and epidemiological context across Mexico. A forum on research priorities hosted by WHO's Special Programme for Research and Training in Tropical Diseases (TDR) decided that the identification of hotspots constitutes a research priority that will help programmes improve the efficiency and effectiveness of resource allocation.^15^ A technical document developed by the Pan American Health Organization (PAHO)^16^ provided a framework, inspired by the Merida findings,^14^ for the implementation of surveillance and control activities using historical city-level case data that are geocoded and spatial analytics to inform resource allocation and vector control activities. As such, Mexico tested the broad applicability of its methodology for hotspot identification as a potential core component of its national strategy. Here, we report findings regarding the spatial correspondence of dengue, chikungunya, and Zika hotspots in nine endemic cities across southern Mexico. These results provide a basis for expanding existing findings about the transmission heterogeneity of dengue and contribute to the development of the PAHO framework to implement urban *Aedes*-borne virus surveillance and control considering arbovirus transmission hotspots.

## Methods

### Study sites

**Figure 1:**
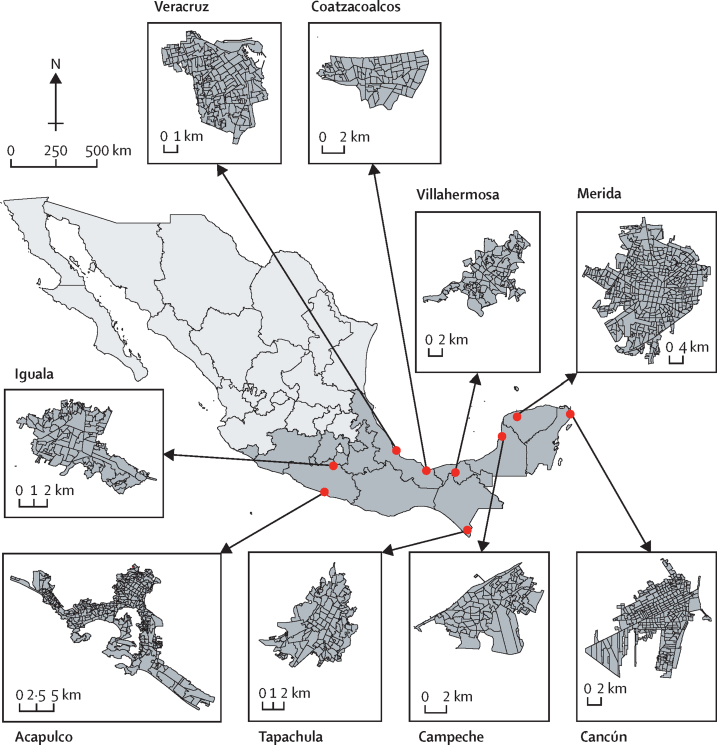
Distribution of census areas in Mexico Figure shows map of Mexico (light grey) showing the states located in southern Mexico (dark grey) and the location and distribution of census areas in the nine cities selected for this study.

We selected nine cities in southern Mexico, which between 2008 and 2016 contributed to 15% of registered dengue cases in the country (figure 1; appendix 2 pp 6–7). Our focus on cities derives from the urban predominance of *A aegypti* and because cases of *Aedes*-transmitted viruses are mostly concentrated in urban areas.^17^ Inclusion criteria for selecting cities were (1) located in southern Mexico (the arbovirus endemic region of Mexico), (2) high burden of dengue cases (ie, more than 5000 cases reported during a 10-year period), and (3) listed as high priority for the Mexican dengue control and prevention programme. Acapulco, Merida, Veracruz, and Cancún each have 0·5–1 million inhabitants. Tapachula, Villahermosa, Campeche, Iguala, and Coatzacoalcos each have over 100 000 but fewer than 500 000 inhabitants (appendix 2 p 6). We used the census tract (Área Geoestadistica Básica [AGEB]) as our unit of spatial analysis. AGEBs measure between 0·20 km^2^ and 0·72 km^2^ and contain between 31 and 48 city blocks (appendix 2 p 6). Acapulco, Merida, Veracruz, and Cancún each have between 263 and 534 AGEBs; Tapachula, Villahermosa, Campeche, Iguala, and Coatzacoalcos each have between 85 and 149 AGEBs (appendix 2 p 6). Given their small size and numbers per city, AGEBs are amenable for spatial analysis and risk stratification.^14^ Basic demographic, environmental, and epidemiological information for each city, including the time series of case reports of dengue, chikungunya, Zika, and all dengue serotypes for 2008–16 are given in appendix 2 (pp 11–16).

### Data management and analysis

Census layers at the AGEB level were accessed from the Instituto Nacional de Estadística y Geografía and the database on probable and confirmed cases of dengue, chikungunya, Zika was accessed from the Sistema Nacional de Vigilancia Epidemiológica (SINAVE) from Mexico's Ministry of Health. This database registers each individual arbovirus case as probable, confirmed, or discarded. The data we used excluded cases that were discarded because of a negative lab diagnosis or the diagnosis of another disease. The data for dengue covered the period 2008–20, the chikungunya data covered 2015 (first detection) and 2016, and the Zika data covered 2016 (first detection). The home address of each probable or confirmed arbovirus case was oded using Google Maps application programming interface (appendix 2 p 17). This detailed geographic information system (GIS) point-layer was summarised by AGEB (to mask individual addresses) as case counts by year. We standardised our counts into a Z score as described by Bisanzio and colleagues.^14^ Maps of all Z scores per AGEB are shown in appendix 2 (pp 18–30) and all spatial data are available online as a geographical dataset.

The Z score values for each disease and year were statistically analysed to detect hotspots using the Getis-Ord Gi*(d) statistic as per Bisanzio and colleagues (appendix 2 pp 3–5).^14^ The Gi*(d) value was corrected for multiple comparisons using a Bonferroni correction that increased the threshold for significance from the classic Z=1·96 to Z=3·71.^14^ A given AGEB was a hotspot if the standardised value of Gi*(d) was greater than or equal to 3·71. If the opposite was true of Gi*(d), the spatial unit was not a hotspot. Dengue hotspots were obtained for each year from 2008 to 2020. Chikungunya hotspots were calculated for 2015 to 2016, whereas Zika hotspots were calculated only for 2016. Transmission hotspots, calculated separately for dengue, chikungunya, and Zika, were identified on the basis of the number of years each AGEB was identified as hotspot.^14^

The concordance between case counts per AGEB for each disease pair was assessed using Kendall's W coefficient,^18^ which was calculated for all possible dengue–chikungunya–Zika combinations. Kendall's W measures the concurrence of two or more quantitative variables and expresses the degree to which the values of the variables is similar or equivalent. W ranges between 0 and 1, where 1 represents perfect concordance. Pairs of variables correspond to one another when the subjects of interest have high values in both the x and y variables (W=1). For all tests, we set alpha as 0·05.

The strength of the hotspot identification methodology to different data structures was assessed by calculating the sensitivity (proportion of AGEBs correctly identified as hotspots) and specificity (the proportion of AGEBs correctly identified as non-hotspots) for a subset of the database compared with the full dataset, as well as comparing two spatial weighting schemes. The subset included all individuals younger than 12 years and older than 70 years (implying two segments of the population that travel less than teenagers and working-age adults). We compared both datasets including a spatial weighting scheme involving immediate neighbours (queen scheme) to a weights matrix set by the inverse of the Euclidean distance of each AGEB centroid. A further validation of dengue hotspot locations involved comparing the location of the hotspot area in 2008–16 with the occurrence of hotspots in an independent (validation) dataset spanning 2017–20. A generalised linear mixed model with a binomial link was parameterised using a measure of the hotspot status of each AGEB (0 = no, 1 = yes) in the validation dataset (2017–20) as a dependent variable, and status of each AGEB with regards to its membership to a historical hotspot area (0 = no, 1 = yes) during 2008–16 as an independent variable. Year was used as a random intercept. To further validate and identify the causes of hotspot occurrence, we contacted heads of vector control of each city and asked then to answer three questions: whether hotspots matched the areas where they have problems controlling dengue, what factors might drive occurrence of hotspots, and what surveillance and control activities can be done in hotspot areas.

All analyses and visualisations were done using R (version 3.4.4 RC) and RStudio (version 1.1.414) with the packages sf, synchrony, sp, spdep, purrr, data.table, ggplot2, tmap, lme4, and GISTools. The R package Caret was used for our specificity estimation.

### Role of the funding source

The funder of the study had no role in study design, data collection, data analysis, data interpretation, or writing of the report.

## Results

From Jan 1, 2008, to Dec 31, 2016, 128 507 clinical dengue cases (mean 14 278 cases per year [SD 8597]) were reported from nine cities, which represented a mean incidence of 43 cases per 100 000 inhabitants (SD 20 cases per 100 000; appendix 2 p 7). Collectively, the nine cities represented 15% of all reported dengue cases in Mexico. Merida accounted for 4% of Mexico's cases, whereas Acapulco, Veracruz, and Villahermosa each accounted for 2%; the remaining cities each accounted for 1% of all reported cases. In all cities, dengue transmission occurs throughout the year with peak incidence occurring during the rainy season (weeks 25–45; appendix 2 p 13). On average, 86% of cases from all cities reported during 2008–16 occurred during the rainy season (appendix 2 p 13). 4752 chikungunya (2015–16)and 25 755 Zika cases (2016) were reported from the nine cities (appendix 2 pp 14–15). For most cities, the seasonal pattern of chikungunya and Zika transmission was similar to that of dengue (appendix 2 pp 13–15). All dengue serotypes were reported throughout the period, with DENV-1, DENV-2 and, more recently, DENV-4 being dominant in Campeche and Merida during 2015 (appendix 2 p 16). A similar proportion of cases of each virus occurred in both sexes (45% in men and 55% in women).

The sensitivity and specificity of each method for estimating hotspots are shown in appendix 2 (p 8). We found strong agreement in most cities between the entire dataset and the subset that only included those aged 12 years and younger and those aged 70 years and older (mean sensitivity 0·77 [SD 0·10], specificity 0·95 [0·02] appendix 2 p 8) and moderate agreement with the subset including Euclidian distance (sensitivity 0·56 [SD 0·17], specificity 0·87 [0·04]; appendix 2 p 8). Age*Euclidean distance were the least sensitive, particularly for Coatzacoalcos (appendix 2 p 8). The irregular shape and size of some census units (figure 1) might explain why Euclidian distance led to reduced sensitivity in the detection of hotspots compared with queen contiguity. Therefore, queen contiguity was preferred over distance-based spatial weighting.

**Figure 2:**
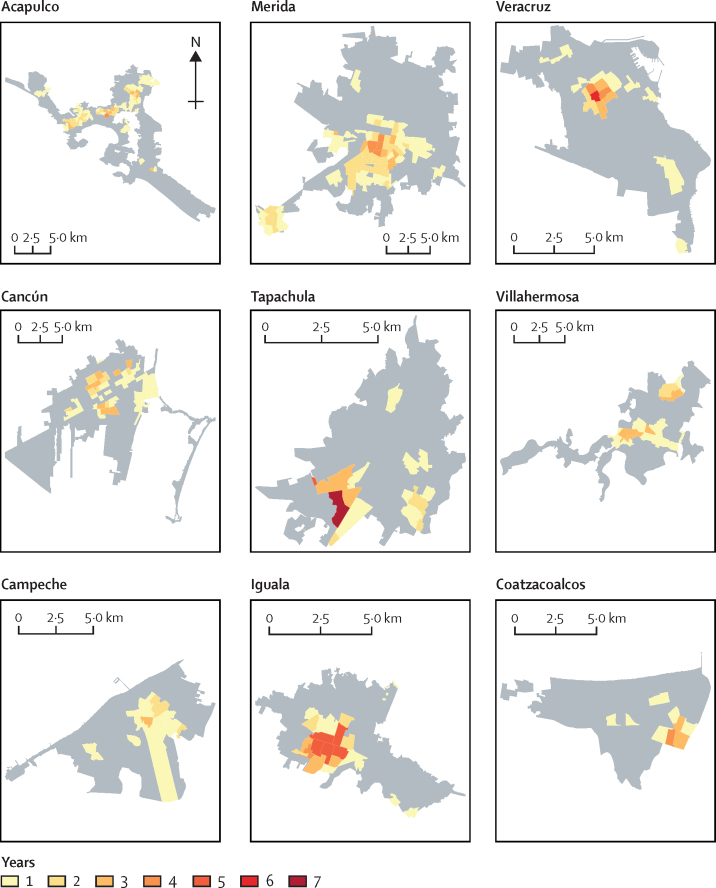
Dengue hotspots in nine cities in Mexico, 2008–16 Colours indicate the number of years each census unit was identified as a statistically significant hotspot using the Getis-Ord Gi*(d) method. Grey areas indicate census units where no significance was detected.

We calculated the Getis-Ord Gi* for each year in the nine cities using queen contiguity and mapped findings as the number of years each AGEB was detected as a hotspot (figure 2). Overall, strong temporal and spatial persistence of dengue case occurrence was observed, with some AGEBs from each city found to be hotspots in all years analysed (figure 2). Across all cities, AGEBs were found to be hotspots for a mean of 3·0 years (SD 2·5; range across cities 1·0–7·0; median 1·0 [IQR 1·0–2·0]). A mean of 17·6% (SD 4·7%; range 10·8–23·2) of the total area and 15·6% (4·1%; 9·1–19·0) of the total number of census tracts of the cities were identified as hotspots (appendix 2 p 9). Between 12·8% and 43·0% of the population of these cities lived in a hotspot (mean 25·6%; SD 9·7; appendix 2 p 9) and the mean percentage of reported *Aedes*-borne disease cases in a hotspot was 32·1% (SD 10·5; range 19·6–50·5; appendix 2 p 9).

**Figure 3:**
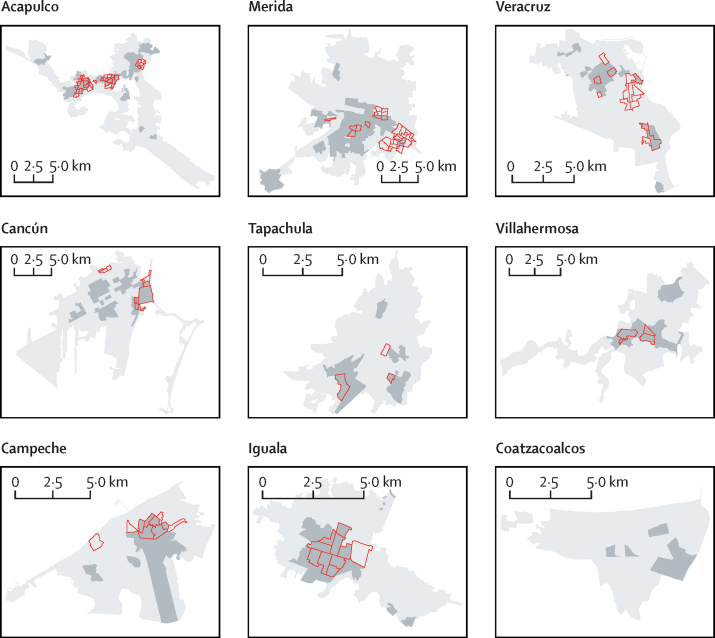
Dengue and chikungunya transmission hotspots in nine endemic cities in Mexico Dengue historical hotspots for 2008–16 are shown in dark grey and red polygons indicate hotpots for chikungunya for 2015–16.

**Figure 4:**
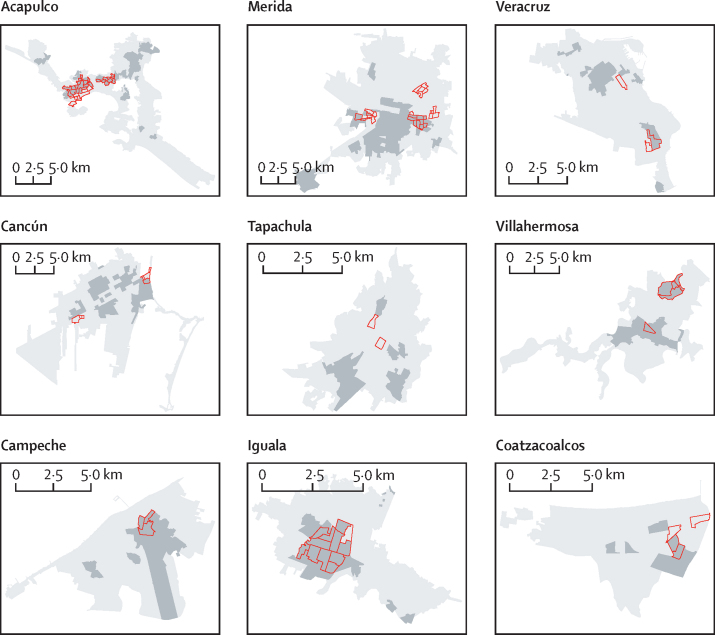
Dengue and Zika transmission hotspots in nine endemic cities in Mexico Dengue historical hotspots for 2008–16 are shown in dark grey and red polygons indicate hotpots for Zika for 2016.

**Figure 5:**
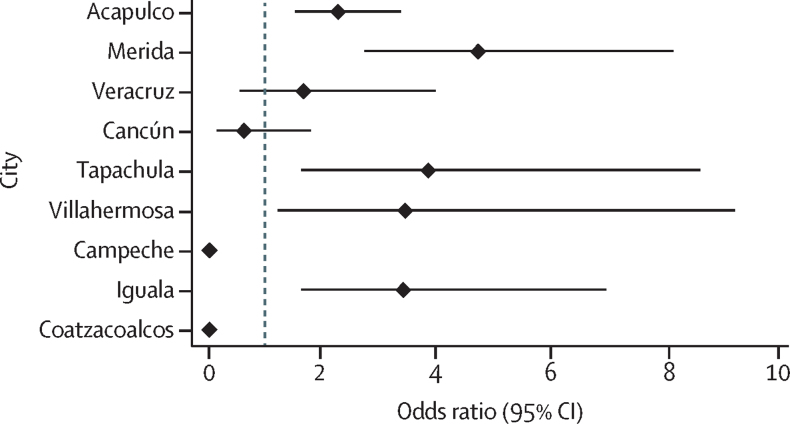
Location of dengue hotspots during 2017–20 compared with the historical hotspot distribution of 2008–16 Results are from a logistic generalised linear mixed model. Values show odds ratios and 95% CIs for each city. Neither Campeche nor Coatzacoalcos had converging models because of their low number of cases (shown as 0).

In most cities, we found a clear overlap between dengue, chikungunya, and Zika hotspots (Figure 3, Figure 4). Across all cities, 61·7% of AGEBs that were identified as hotspots of Zika overlapped with historical dengue hotspots (Figure 3, Figure 4). For chikungunya, overlap reached an average of 53·3% for both years of chikungunya transmission (and 60·5% for 2016 only; Figure 3, Figure 4). Iguala (40·7%), Villahermosa (36·8%), and Acapulco (33·0%) were the cities with the most overlap between historical dengue and chikungunya–Zika, whereas Cancun (12·3%) and Tapachula (14·3%) were the cities with the least overlap (possibly due to the intensive movements of migrants, refugees, and tourists to these areas that have led to rapidly growing suburban areas; Figure 3, Figure 4). When looking at the distribution of Z scores by AGEB, the Kendall's W calculated agreement between pairs of diseases, ranging between 0·55 and 0·92 (appendix 2 p 10), with a mean W of 0·70 (dengue and chikungunya 2015), 0·72 (dengue and chikungunya 2016), 0·84 (dengue and Zika 2016), 0·70 (Zika 2016 and chikungunya 2015), 0·76 (Zika 2016 and chikungunya 2016), and 0·65 (chikungunya 2015 and chikungunya 2016; appendix 2 p 10). Significance of W was detected in 49 of 54 virus–city comparisons (appendix 2 p 10). Further, strong and significant agreement in the location of dengue hotspots in 2008–16 and in 2017–20 was quantified by generalised linear mixed models in Acapulco, Merida, Tapachula, Villahermosa, and Iguala (figure 5). Veracruz showed a positive but non-significant association, whereas Cancun showed a negative but non-significant association (figure 5). The distribution of cases in Campeche and Coatzacoalcos was too low to detect hotspots (figure 5; appendix 2 p 30).

Heads of vector control for each city reported high agreement between the hotspot areas and the areas historically contributing with most dengue cases in seven out of nine cities (77·8%), whereas in two cities the agreement was partial due to rapid demographic changes observed in recent years (table). For instance, in Tapachula and Villahermosa areas in the periphery (new informal urbanisations to house a large migrant population) were not available because AGEBs and could not be mapped. Water shortage, the need to accumulate water in the dry season, violence, and other social factors were identified by heads of vector control as root problems in hotspot areas (table).

**Table:** Agreement of hotspot analysis with the areas identified as problematic for arbovirus transmission

	**Does the hotspot area match with areas you identify as problematic (yes, partially, no)?**	**What local conditions determine the occurrence of hotspots (ecological, environmental, infrastructure, social, or other determinants)?**	**What surveillance or control activities could you implement in your city considering the presence of hotspots?**
Acapulco	Yes: most problematic neighbourhoods were included—eg, Zapata, Renacimiento, Coloso, and Colsio	Infrastructure: water shortage and irregular delivery of water forces people to accumulate water in large containers that become habitats for *Aedes aegypti* Social: violence and crime in some areas (only 10% of area can be safely accessed) limit coverage by vector control teams Other: little community engagement in removal and control of larval habitats and fears of violence reduce access of vector control personnel in residents’ houses	Surveillance: conduct entomo-virologic surveillance in hotspots (infection in mosquitoes) Control: integrated vector management, including larval control, residual insecticide application, monthly spatial spraying, and routine impact evaluation
Mérida	Yes: most problematic areas coincide with hotspots	Sociodemographic: high population density areas with different levels of education Ecological: high prevalence of infested houses with a diversity of breeding-sites during the rainy season, dominated by plastic containers (buckets) that people accumulate and are difficult to eliminate Environmental: premises with large and vegetated patios (shady), new *vs* old houses of low-middle class built in the 1950s, many with cement elevated water tanks that are deteriorated and serve as habitats for *A aegypti*; closed houses propose challenges for larviciding and control Infrastructure: catch basins in the streets serve as mosquito habitats during dry season	Surveillance: enhanced surveillance with ovitraps and active search for cases Control: preventive vector control before transmission season (education campaigns, source reduction, indoor residual spraying) and case response using truck-mounted ultra-low volume and indoor space spraying. Other: house and public infrastructure improvements (mosquito screens, anti-mosquito water tanks and catch basins)
Veracruz	Yes: the hotspot area is the zone where historically cases accumulate	Infrastructure: although 98% of Veracruz has piped water access, during the dry season water supply is irregular (water is provided to households once a week because river levels are low) and people have to accumulate water in large containers Social: gaining access to houses is difficult because residents work long hours, leaving approximately 30% of premises unable to be controlled	Surveillance: entomo-virological surveillance in hotspot areas throughout the year would allow detecting early transmission Control: an intervention package during pre-transmission and during transmission seasons could be deployed (including health promotion, epidemiological surveillance, and vector control)
Cancún	Yes: the hotspots match our problem areas	Environmental: in many houses in the hotspot, buildings are multi-story and obtain their water using shared cisterns, making them key *A aegypti* breeding habitats; many cisterns are broken or not well kept, allowing for *A aegypti* entry and egg laying Social: violence limits access of vector control personnel into the areas; low socioeconomic status prevails, and houses are often crowded with large numbers of families	Surveillance: ovitrap surveillance is important but by knowing hotspots we could implement preventive activities using health promotion for the elimination of breeding habitats and improve health awareness Control: we can more aggressively use insect-growth regulators in hotspot areas; other actions involve thermal fogging of alleys and areas of difficult access, and application of larvicide using portable sprayers to minimise entrance to houses
Tapachula	Partially: hotspot areas are problem zones but areas in the periphery of the city that were not included in the analysis also contribute with many cases	Environmental: sustained population growth with poor planning has led to informal settlements and many larval habitats and land tenure is not stable Social: this is a border city and migration and mobility can introduce viruses	Surveillance: strengthen primary health care to enhance search for cases in hotspot areas Control: integrate vector control actions sequentially (source reduction, larval control, residual spraying, and spatial spraying) and in coordination with epidemiological surveillance
Villahermosa	Partially: hotspot areas are problem zones but areas in the periphery of the city that were not included in analysis also contribute with many cases	Ecological: small plastic containers, followed by large tanks (>200 L), are the most common *A aegypti* habitats Environmental: houses lack screens and are of poor condition Infrastructure: deficient water supply and water is accumulated in containers for personal use Social: informal settlements aggravate the situation, and the prevalence of crime makes cases difficult to control	Surveillance: more active case detection in hotspot areas will help with responding to cases; more emphasis of ovitrap sampling in hotspot areas to detect mosquito numbers; entomo-virological surveillance can be implemented in hotspots Control: immediate larval control, followed by indoor residual spraying at high coverage, and ultra-low volume spraying once cases are detected
Campeche	Yes: hotspots match our problem areas	Ecological: big drums and cisterns are the typical *A aegypti* breeding habitat, together with plastic buckets and small plastic containers; patios are large and have abundant tree cover Environmental: these areas started as irregular (illegal) settlements that have improved in infrastructure and services, but do not have piped water inside the home; toilet flush occurs with buckets, and large drums and containers are needed to store water on a daily basis as well as during the long dry season Infrastructure: no elevated water storage tanks limits water storage capability and forces people to store water in their patio or house Social: these are settlements inhabited by people with low socioeconomic status, some of whom come from other states and have informal jobs; neighbourhoods are also known for their problems in safety and crime is a problem for control personnel and residents	Surveillance: entomological surveillance with ovitraps is done; the hotspot areas would benefit from entomo-virological surveillance to detect early transmission Control: actions that each resident can do would be applicable, encouraging better caring of their patio and reduction of larval habitats; larval (long lasting) control of large water storage containers would be cost-effective
Iguala	Yes: hotspot area contributes approximately 40% of all cases and is where government offices and areas of large human movement are concentrated	Infrastructure: deficient water access (supply once every 2 months) forces people to have large water storage containers Other: little community involvement in control of larval habitats; householders limit access of health personnel into their houses	Surveillance: strengthen programme by incorporating entomo-virological surveillance in hotspots; quantification of main larval habitats by city block to better target larval control Control: integrated actions involving larval control, residual spraying and ultra-low volume spraying
Coatzacoalcos	Yes: hotspot area contributes large number of cases to the city	Environmental: urban houses with regular water supply; typical larval habitats involve small diverse plastic containers Infrastructure: long dry season reduces waterbodies and exposes larval habitats; other small containers have water for extended periods of time Social: violence in these areas limits the proper access of health personnel and regular control actions	Surveillance: entomo-virological surveillance would enhance our ability to detect viruses before and during transmission season Control: early control and integrated vector management actions would include health promotion, risk assessment, and epidemiological surveillance; residual spraying could be done more effectively, and combined with larval control before the peak transmission; during emergencies, ultra-low volume spraying and thermal fogging

Information was provided by heads of vector control of each city with regards to the agreement of hotspot analysis with the areas they identified as problematic for arbovirus transmission, as well as the drivers behind the occurrence of hotspot areas and some possible surveillance and control actions that can be taken to control Aedes-transmitted viruses using the hotspot framework.

## Discussion

In the digital era of big data, ministries of health have increasingly been creating systems that collect, accumulate, and protect digital information about the occurrence and geographical location of health records. Following this tendency, in 2008, Mexico created a platform where the SINAVE stores epidemiological information about dengue and, beginning in 2015, chikungunya and Zika. Databases generated by the SINAVE platform were initially used to quantify the long-term (2008–16) trends in dengue transmission and chikungunya and Zika introductions in Merida, Mexico.^14^ We have broadened the scope of these analyses to include nine cities, and confirmed the generalisability of methods for identifying urban hotspots of arbovirus transmission.

Hotspots were found in all cities, regardless of the population size. Variability among cities occurred in the proportion of reported arbovirus cases within hotspots; Iguala accumulated approximately 50% of cases in approximately 23% of its area whereas Veracruz had 18% of cases in 11% of its area. Unfortunately, data limitations prevented analysing the occurrence of hotspots by dengue serotype. Heads of vector control confirmed hotspots as problem areas in all cities. Irregular water supply (which leads to water accumulation in large and diverse containers), violence and crime, as well as lack of trust from communities (which limits access into houses by vector control teams) were identified as important determinants of transmission risk in hotspots. Migrant and mobile populations and informal settlements were also mentioned as contributors in some cities. The combination of strong intra-city heterogeneity and disease overlap provide evidence for the redesign of *Aedes*-borne disease surveillance and control strategies in those cities. Our findings support the policy statement by WHO's TDR indicating that the identification of high risk transmission areas—or hotspots—is an urgent need for *Aedes* control programmes.^15^ PAHO has developed a framework for risk stratification using thxotspot detection methods outlined here^16^ and is training public health personnel in a weeklong workshop transferring the use of GIS and spatial statistics to member countries.

Other study limitations include the fact that only symptomatic cases were mapped, preventing an estimate of the contribution of hotspot areas to the absolute burden of infections in each city. Furthermore, because we mapped cases to their residential address, we might have failed to identify out-of-home locations where infections might have occurred. Although in our sensitivity analysis that focused on a narrow age group we attempted to assess the potential confounding of exposure outside the home, we are aware that having mobility information for each case would have substantially strengthened our findings.^19^ An extended explantation of the limitations is given in appendix 2 (p 5).

We applied a simple and robust methodology for the detection of spatial patterns of cases in data aggregated at the census-tract level. The calculation of Z scores of case counts, combined with the use of the local spatial statistic Getis-Ord Gi*(d) unveiled consistent spatial trends that, in Merida, were validated with an independent longitudinal dengue sero-survey.^14^ Because each dengue outbreak might lead to a different spatial pattern of cases, the validity of our approach relies on the analysis of multiple years (ideally more than 5 years) to capture enough variability in transmission patterns. Including novel virus introductions (eg, chikungunya and Zika) in analyses can further enhance the identification of high-risk areas, given their transmission is independent from the dengue immunity status of the human population and more influenced by *A aegypti* distribution and human–mosquito contacts. Furthermore, we used an independent dataset and statistically validated the occurrence of hotspots in five cities, with two cities (Campeche and Coatzacoalcos) not having enough cases to detect hotspots.

Over 9 years, the maximum number of times that a tract was a hotspot for dengue was seven, with tracts displaying an average temporal persistence of 3 years. Temporal persistence of dengue transmission hotspots has been documented, using various statistical methods across the pathogen's distribution range.^14^, ^20^, ^21^, ^22^, ^23^, ^24^, ^25^, ^26^ Underlying the concept of temporally persistent hotspots is the notion that surveillance and control can be more efficient and effective if these methods account for this heterogeneity in risk.^13^ Our analyses show that in some cities, up to 50% of the reported cases are concentrated in approximately 30% of the area (also reported by Bisanzio and colleagues^14^). This finding is particularly valuable information for prevention and control programmes for *Aedes*-borne disease, if used programmatically. For instance, after Zika introduction, Merida used the maps published by Bisanzio and colleagues^14^ to implement PCR-based virus surveillance in *A aegypti* collected in houses from the hotspot area. In 2017, entomo-virological surveillance detected DEN-3-positive mosquito pools (a novel introduction) within the hotspot area (Palacio-Vargas J, unpublished). An aggressive vector control campaign across the hotspot area involving indoor space spraying, larviciding, and community education was deployed. No human cases of DENV-3 were detected in Merida during that year or subsequent years, indicating the value of focusing virus surveillance of *A aegypti* within hotspot areas. Heads of vector control identified the value of focusing entomo-virological surveillance in hotspots.

How to deploy *A aegypti* control is crucial to the success or failure of an intervention. In areas prone to sporadic *Aedes*-borne virus introductions (eg, Europe, USA, or Australia), integrating epidemiological surveillance platforms with contact tracing can help deploy indoor spraying and contain dengue outbreaks.^19^, ^27^ In endemic areas, the high vector density, low coverage of health services, poor health-seeking behaviour of communities, inapparent infections, and insuffcient budgets and personnel, can inhibit the effect of reactive approaches, particularly during outbreaks. Evidence from modelling studies show that, in endemic areas with seasonal dengue transmission, preventive *A aegypti* vector control (ie, before the regular transmission season) could substantially increase the effectiveness of interventions in comparison with reactive control.^28^, ^29^ Preventive and long-lasting actions could thus improve vector control efficacy without the need for substantial additional resources. One of the limitations of preventive control, however, is the fact that in most cases doing so would not be logistically feasible over an entire city. Our analyses and evidence of hotspot presence and persistence from other studies provides a logical framework for guiding the prioritisation of preventive control actions. Hotspot areas, given their persistence and important role during outbreaks, can be targeted with preventive and long-lasting interventions before the peak transmission period. Our consultation with heads of vector control for each city identified options that they suggest as relevant within hotspots, such as the implementation of entomo-virological surveillance and preventive control in hotspots using larviciding, indoor residual spraying, or a combination of approaches. The acute issues experienced in cities with water supply also provide evidence for potential intersectoral environmental management solutions, with important benefits for urban sustainable development. Novel area-wide strategies such as the sterile insect technique or *Wolbachia*-mediated approaches could also target hotspots for their initial roll-out, because these areas might experience the greatest benefit in the short term compared with releasing mosquitoes in lower-risk zones. A feasible control strategy could entail proactive control in hotspots and prompt reactive actions in non-hotspot areas.

Estimates regarding the economic effects of dengue in Mexico in 2010–16 showed that 40–56% of costs incurred are related to investments in vector control.^9^, ^10^ These calculations assumed a model of universal (non-focused) vector control without considering the spatial heterogeneity of dengue. Applying a focused strategy of anticipatory control and prevention to hotspots could have a direct effect in reducing dengue burden and the indirect costs related to hospitalisation, laboratory diagnostics, purchase of household insecticides, and programmatic vector control. Future research should focus on the field assessment of the costs, benefits, and epidemiological effects of spatially focused interventions in *Aedes*-borne disease hotspots.^14^ Potential changes in the distribution of hotspots, due to targeted interventions or changes in demographic or epidemiological trends, would have to be routinely investigated and addressed in an adaptive and iterative process.^16^ Our use of 2017–20 as a validation dataset shows that this process is both possible and useful. Devising a rational approach for intervention delivery to prevent *Aedes*-borne viruses could greatly benefit from the use of risk stratification within cities.

## Data sharing

Original files, including de-identified case counts (standardised) for each city (aggregated at the AGEB level) are available upon publication of this Article as a shapefile geographical dataset from https://doi.org/10.17632/m6nvnzgzdt.1.

## Declaration of interests

We declare no competing interests.

## Supplementary Materials

Spanish translation of the abstract
