# Supplementary material for: Identifying urban hotspots of dengue, chikungunya, and Zika transmission in Mexico to support risk stratification efforts: a spatial analysis
Source: Lancet Planet Health. 2021 May 5;5(5):e277–85. doi: 10.1016/S2542-5196(21)00030-9 (PMC8114339; doi:10.1016/S2542-5196(21)00030-9)
Supplement: Spanish translation of the abstract [file mmc1.pdf]

THE LANCET
Planetary Health

Supplementary appendix 1

This appendix formed part of the original submission. We post it as supplied by the authors.

Supplement to: Dzul-Manzanilla F, Correa-Morales F, Che-Mendoza A, et al. Identifying urban hotspots of dengue, chikungunya, and Zika transmission in Mexico to support risk stratification efforts: a spatial analysis. *Lancet Planet Health* 2021; **5:** e277–85.

This translation in Spanish was submitted by the authors and we reproduce it as supplied. It has not been peer reviewed. *The Lancet*'s editorial processes have only been applied to the original in English, which should serve as reference for this manuscript.

Los autores nos proporcionaron esta traducción al español y la reproducimos tal como nos fue entregada. No la hemos revisado. Los procesos editoriales de *The Lancet* se han aplicado únicamente al original en inglés, que debe servir de referencia para este manuscrito.

Resumen

Antecedentes

El control efectivo *de Aedes aegypti* se ve desafiado, en parte, por la dificultad de lograr una extensa y eficaz cobertura de intervenciones. Para maximizar el impacto del control de vectores, se podrían identificar y priorizar zonas con alto y persistente número de casos de enfermedades transmitidas por *Aedes*. En este estudio reportamos el hallazgo de focos persistentes (hotspots) de enfermedades transmitidas por *Aedes* en ciudades del sur de México.

Métodos

Casos geocodificados de dengue (DEN), chikungunya (CHIK) y Zika (ZIK) procedentes de nueve ciudades mexicanas endémicas fueron agrupados al nivel de distrito censal. Con el objetico de identificar espacialmente hotspots de cada enfermedad en cada ciudad, datos anuales de casos fueron analizados con el estadístico Getis-Ord *Gi*(d).* El coeficiente Kendall *W* calculó la convergencia en la distribución de cada virus.

Resultados

Entre 2008-2016 se notificaron un total de 128.507 casos clínicos de DEN, 4.752 CHIK y 25.755 ZIK. Todas las ciudades mostraron evidencia de heterogeneidad de transmisión, con un promedio del 17,6% de su área identificada como focos de infección persistentes. Los hotspots representaron el 25·6% (rango, 13-43%) de la población y todos los casos de enfermedad transmitida por *Aedes* notificados representaron el 32·1% (rango, 20-51%). Se encontró una superposición entre los hotspot para DEN-ZIK del 62% y del 53% para DEN-CHIK. También se encontró una asociación significativa entre los hotspot de DEN reportados entre el 2008 y el 2016 con los hotspots reportados durante el 2017 y el 2020 en cinco de las nueve ciudades. Los jefes de control de vectores de cada ciudad confirmaron que los hotspot detectados son zonas problemáticas por su alta transmisión de arbovirus.

Interpretación

Este estudio proporciona evidencia de la superposición de enfermedades transmitidas por *Aedes* dentro de hotspots y provee un marco metodológico para la estratificación del riesgo de transmisión de arbovirus dentro de las zonas urbanas, la cual podría servir de guía para la implementación de estrategias de vigilancia y control de vectores.
