# Supplementary material for: Identifying urban hotspots of dengue, chikungunya, and Zika transmission in Mexico to support risk stratification efforts: a spatial analysis
Source: Lancet Planet Health. 2021 May 5;5(5):e277–85. doi: 10.1016/S2542-5196(21)00030-9 (PMC8114339; doi:10.1016/S2542-5196(21)00030-9)

THE LANCET
Planetary Health

Supplementary appendix 2

This appendix formed part of the original submission and has been peer reviewed. We post it as supplied by the authors.

Supplement to: Dzul-Manzanilla F, Correa-Morales F, Che-Mendoza A, et al. Identifying urban hotspots of dengue, chikungunya, and Zika transmission in Mexico to support risk stratification efforts: a spatial analysis. *Lancet Planet Health* 2021; **5:** e277–85.

Supplementary Appendix for

Identifying urban hotspots of dengue, chikungunya and Zika transmission in Mexico to support risk stratification efforts

Felipe Dzul-Manzanilla, Fabián Correa-Morales, Azael Che-Mendoza, Jorge Palacio-Vargas, Gustavo Sánchez-Tejeda, Jesus F González-Roldan, Hugo López-Gatell, Adriana E. Flores-Suárez, Hector Gómez-Dantes, Giovanini E. Coelho, Haroldo S. da Silva Bezerra, Norma Pavia-Ruz, Audrey Lenhart, Pablo Manrique-Saide, Gonzalo M. Vazquez-Prokopec

Centro Nacional de Programas Preventivos y Control de Enfermedades (CENAPRECE), Ministry of Health, Mexico (F Dzul-Manzanilla, MSci, F Correa-Morales, MSci, G Sánchez-Tejeda, MD)**.**
Collaborative Unit for Entomological Bioassays (UCBE), Campus de Ciencias Biologicas y Agropecuarias, Universidad Autonoma de Yucatan, Merida, Yucatan, Mexico (A Che-Mendoza PhD, Prof P Manrique-Saide PhD).
Servicios de Salud de Yucatan, Merida, Yucatan (J Palacio-Vargas MSci).
Sociedad Mexicana de Salud Publica, Mexico City, Mexico (JF González-Roldan MD).
Subsecretaria de Prevencion y Promocion de la Salud, Mexico City, Mexico (H López-Gatell PhD).
Facultad de Ciencias Biologicas Universidad Autónoma de Nuevo Leon, San Nicolas de los Garza, Nuevo Leon, Mexico (Prof AE Flores-Suárez PhD).
Health Systems Research Center, National Institute of Public Health, Cuernavaca, Mexico (Prof H Gómez-Dantes MD).
Pan American Health Organization (PAHO), Department of Communicable Diseases and Environmental Determinants of Health, Washington, DC, USA (GE Coelho PhD, HS. da Silva Bezerra MSc).
Centro de Investigaciones Regionales Hideyo Noguchi, Universidad Autonoma de Yucatan, Merida, Mexico (N Pavia-Ruz MD).
Centers for Disease Control and Prevention, Atlanta, GA, USA (A Lenhart PhD**).**
Department of Environmental Sciences, Emory University, Atlanta, GA, USA (GM Vazquez-Prokopec PhD**).**

Index

Content **Page#**

Basic description of each city…………………………………………………….3
Extended description of statistical analyses……………………………………..3
Description of statistical analyses………………………………………………..5
Study Limitations…………………………………………………………………5
Supplementary Tables
Table S1………………………………………………………………………6
Table S2………………………………………………………………………7
Table S3………………………………………………………………………8
Table S4………………………………………………………………………9
Table S5………………………………………………………………………10
Supplementary Figures
Figure S1……………………………………………………………………..11
Figure S2……………………………………………………………………..12
Figure S3……………………………………………………………………..13
Figure S4……………………………………………………………………..14
Figure S5……………………………………………………………………..15
Figure S6……………………………………………………………………..16
Figure S7……………………………………………………………………..17
Figure S8……………………………………………………………………..18
Figure S9……………………………………………………………………..19
Figure S10……………………………………………………………………20
Figure S11……………………………………………………………………21
Figure S12……………………………………………………………………22
Figure S13……………………………………………………………………23
Figure S14……………………………………………………………………24
Figure S15……………………………………………………………………25
Figure S16……………………………………………………………………26
Figure S17……………………………………………………………………27
Figure S18……………………………………………………………………28
Figure S19……………………………………………………………………29
Figure S20……………………………………………………………………30

Basic description of each city

The nine cities include commercial, educational, touristic, economical, and cultural centers with internal, inter-state, and international population flows. In the localities we examined, precipitation is assessed on the basis of two seasons: a dry season, occupying the first 20 weeks of the year (January to May), which is characterized by a limited amount of rainfall, and a wet season which is characterized by increased rainfall and occupies the 20th-40th weeks of the year (mid-May to October); the remainder of the year sees intermittent rainfall (Figure S1) (http://smn.cna.gob.mx/es/climatologia/informacion-climatologica). The wet season typically experiences from 934·3 to 2431·4 mm of water accumulation in the localities of this study, while the dry season usually experiences 65·2 to 670 mm of water accumulation. The minimum temperature falls between 17·9ºC and 22.8ºC during the wet season and between 18·7ºC a 24·4ºC during the dry season (Figure S2). The maximum temperature ranges between 28·8°C and 35·3°C during the wet season and 29·6°C and 32·9°C during the dry season (Figure S2). Considering the seasonality in temperature and rainfall, coupled with the distribution of dengue cases, all cities had an arbovirus transmission period spanning from weeks 25 and 45 (Figure S3). On average, 86% of cases from all cities were reported during the transmission period. Some notable exceptions to this dengue transmission season include the year 2012 in Villahermosa and the years 2011 and 2009 in Merida. Chikungunya transmission show a less marked seasonality, driven by the time when the virus was first introduced and detected in each city (Figure S4). For Acapulco, Villahermosa, y Cancun and Coatzacoalcos, a large number of cases occurred outside the regular dengue transmission season, particularly in 2016 (consequence of an extended outbreak that transitioned from 2015 into 2016) (Figure S4). Zika, on the other hand, behave more similarly to dengue in its seasonality, with the exception of Tapachula that presented most reported cases within the first 10 weeks of 2016 (Figure S5). Particularly for dengue, a small fraction (20%) of cases were typified to the serotype level (Figure S6). The trend in dengue serotype occurrence in the nine cities shows alternated dominance of DEN-1 and DEN-2 until 2013, when DEN-4 was introduced and had variable dominance in cities such as Campeche, Merida and Acapulco (Figure S6). Interestingly, not all cities were equally invaded by DEN-4, indicating differential circulation of this serotype in comparison with DEN-1 and DEN-2.

Extended description of statistical analyses (Local Getis-Ord G* spatial statistic)

When one performs spatial analyses of rare diseases in small area units, classical measures such as incidence are prone to the 'small numbers problem' in that a very small number of cases can significantly inflate rates, leading to deceiving conclusions about the true spatial pattern. Using raw cases (case counts) has the issue of variability between years; spatial dynamics could be driven by one or a few years with large number of cases. Creating a standardized measure of case distribution using z-scores addresses some of the numeric biases that emerge when using incidence or case counts. Some advantages of using z-scores are: 1) it allows for a measure that is comparable among cities and years, no matter their absolute case count; 2) given analyses are done in small areas with similar population size, the z-score is a measure of transmission intensity that is more relevant to decision makers than incidence (areas with high z-score indicate

census units with large number of cases for that year, no matter the total case load); 3) z-scores are easily calculated even in the presence of inaccurate census data for the number of inhabitants per census tract (given population in urban census tracts can vary dramatically over a decade, we avoided using incidence based on an unreliable measure of population at risk). As for disadvantages, the main one is that z-scores are unit-less and difficult to interpret as single values. One may not know true intensity of transmission between years by only using z-scores, but can compare a map of z-scores with one of case counts to retrieve such information. Other disadvantages are that z-score does not account for population imbalances if census tracts vary markedly (several orders of magnitude). This may be less of an issue if the goal is to respond to areas that have a significant burden (not incidence) of cases.

Our procedures for hotspot detection are shown in Figure S7. Briefly, we geocoded the original dataset from SINAVE including residential addresses using Google API, obtaining an 83% accuracy at the house or street intersection levels. Those cases correctly geocoded were then joined with a shapefile of AGEBs (census units) to obtain the count of the number of cases per census unit. The counts per year in the attribute table of the AGEB shapefile were standardized using the z-score equation shown Bisanzio et al.[14] In R, we generated the adjacency matrix (neighborhood matrix) for all AGEBS and used it (together with the z-scores per year) to calculate the Getis-Ord Gi*(d) statistic (Figure S7).

The local Getis-Ord G* spatial statistic (Getis & Ord, 1992, 1995) was calculated by the following formula:

$$G_i^*(d) = \frac{\sum_j w_{ij}(d)x_j - w_i^* \mu}{s\sqrt{\frac{nS_{1i}^* - w_i^{*2}}{n-1}}}$$

In this formula, $w_{ij}(d)$ is i, j is the spatial weights matrix with a value of 1 if the spatial unit j is adjacent or within the inverse of the Euclidean distance of the spatial unit i; otherwise the value of j is 0. $w_i^* = \sum w_{ij}(d)$ when w_{ii} is included; $S_{1i}^* = \sum w_{ij}^2$; x_j is the number of cases in the spatial unit j for each arbovirus and year; n, μ & s represent respectively the number of spatial units, the mean, and the standard deviation of the number of cases.

The areas j adjacent to the area i of $w_{ij}(d)$ were defined using two foci. In the first case, the adjacent areas were defined by the "queen" scheme in which the spatial unit i shared all of its borders wiht the spatial units j. With the second focus, the inverse of the Euclidean distance was used to define the threshold of the distance within which the centroids of the spatial units j are adjacent to the centroids of the spatial unit i. In both cases, the the spatial unit j was given a value of 1 if it was adjacent to the spatial until i or if it was within the inverse of the spatial unit i's Euclidean distance; otherwise, j was 0.

The value of $G_i^*(d)$ can be: values similar to 0, values less than 0 (negative), or values greater than 0 (positive). In the first case, values close to 0 indicate that the number of cases in the adjacent spatial units j are randomly distributed by the spatial unit i. On the other hand, if the positive or negative values of $G_i^*(d)$ are statistically non-zero, it is interpreted that the number of cases are not distributed randomly and that the cases have an aggregated distribution. It is

determined that a spatial unit is a hotspot if the values are positive and larger than a threshold st for the z-value of the test statistic, and a coldspot if the values are lower than the negative value of the threshold. With infectious disease, the identification of hotspots is typically more important than that of coldspots.

Description of statistical analyses (Sensitivity, specificity)

To measure the performance of the different methodological strategies of hotspot identification, sensitivity and specificity were calculated. For the calculation of these parameters, a confusion matrix was constructed, which is a 2 x 2 table where the cells indicate the number of space units that were correctly identified as hotspots (TH, True hotspots) or non- hotspots (TNH, True non-hotspots) and the number of space units that were not correctly identified as hotspots (FH, False hotspots) or non-hotspots (FNH, False non-hotspots) with both methodologies

Test group	Reference group	
	hotspots	non-hotspots
hotspots	TH	FNH
non-hotspots	FH	TNH

Sensitivity (proportion of spatial units correctly identified as hotspots) and specificity (proportion of spatial units correctly identified as non-hotspots) were calculated using the following formulas:

$$Sensitivity = \frac{TH}{(TH + FH)}$$

$$Specificity = \frac{TNH}{(TNH + FNH)}$$

Study Limitations

Like all studies based on epidemiological data collected by passive surveillance, this study has limitations that do not invalidate the results but that need to be considered and discussed. Surveillance systems only detect symptomatic cases from individuals seeking medical care, and the adult population in endemic areas often fails to obtain healthcare in the presence of mild symptoms. In addition, symptomatic dengue is manifested through fever and can be confused with many similarly manifesting diseases, resulting in clinical false positives. Something else to consider: the manifestation of dengue varies along a spectrum—sometimes it causes fever and sometimes it is asymptomatic. The passive surveillance system also registers the address of the residence of symptomatic patients and such information is the one used to geocode each case, without consideration of the fact that a large proportion of infections may occur in places other than the home. In order to partially address these challenges, we ran analyses both in the full dataset and in a subset including children (under twelve years of age) and seniors (over 70 years of age). These age groups could be considered less mobile more vulnerable to infection in their household and more likely to seek healthcare. When comparing our results across the full dataset

and its subsets, small differences in accuracy and specificity were detected, providing evidence that our ability to detect hotspots is robust to changing assumptions of mobility and reporting.

Supplementary Tables

Table S1. Demographic and spatial attributes of each city selected for this study.

Locality, State	Total 2010 population	Population Size strata*	Urban Area (km^2)	Total city blocks	Census tracks		
					No. units	Average area (km^2)	Average number of city blocks
Acapulco, Guerrero.	673,479	0.5-1M	137•1	15,451	463	0•30	38•7
Mérida, Yucatán.	906,722	0.5-1M	257•6	15,404	534	0•48	30•8
Veracruz, Veracruz.	577,166	0.5-1M	73•9	10,052	263	0•28	45•1
Cancún, Quintana Roo.	633,648	0.5-1M	132•8	10,480	315	0•42	37•7
Tapachula, Chiapas.	208,975	<0.5M	44•7	4,129	143	0•31	39•1
Villahermosa, Tabasco.	382,091	<0.5M	76•4	4,552	106	0•72	47•8
Campeche, Campeche,	221,500	<0.5M	51•3	3,733	116	0•44	35•4
Iguala, Guerrero.	118,468	<0.5M	29•9	3,173	149	0•20	25•8
Coatzacoalcos, Veracruz.	246,562	<0.5M	42•6	3,461	85	0•50	46•6

*Separation of cities based on their population size: 0.5-1M are cities with population between 500,000 and 1,000,000 whereas <0.5M are cities with population below 500,000.

Tabla S2. Number of symptomatic dengue cases reported to Mexico's passive surveillance system.

City	Strata*	2008	2009	2010	2011	2012	2013	2014	2015	2016	2017	2018	2019	2020	Total
**Acapulco**	0·5-1M	1320	3616	2137	336	3397	1276	843	1954	1457	22	6	92	79	16,535
**Mérida**	0·5-1M	673	3879	1811	6446	8080	2530	1772	6229	2751	6	2	46	28	34,253
**Veracruz**	0·5-1M	591	825	247	246	5101	2064	2691	3347	2080	16	76	148	45	17,477
**Cancún**	0·5-1M	481	467	1421	2761	1650	2670	699	1921	763	6	2	208	26	13,075
**Tapachula**	<0·5M	495	240	152	201	1720	975	1146	786	298	647	390	153	13	7,216
**Villahermosa**	<0·5M	343	3100	331	123	1485	5652	312	2650	2602	6	2	33	15	16,654
**Campeche**	<0·5M	31	332	521	757	1378	556	337	2805	1515	0	0	15	7	8,254
**Iguala**	<0·5M	1338	433	1800	240	1397	185	108	814	632	10	2	6	8	6,973
**Coatzacoalcos**	<0·5M	706	2135	77	44	1062	2993	238	1886	1044	8	35	55	8	10,291

*Separation of cities based on their population size: 0·5-1M are cities with population between 500,000 and 1,000,000 whereas <0·5M are cities with population below 500,000.

Table S3. Sensitivity and specificity of different approaches used to quantify hotspots of dengue in each city. The reference in all calculations is the complete dataset.

City			Age <= 12 & <= 70 years				Euclidean distance				Age <= 12 & <= 70 years * Euclidean distance		
	Strata*		sensitivity	specificity			sensitivity	specificity			sensitivity	specificity	
**Acapulco**	0·5-1M		0·67	0·96			0·61	0·87			0·68	0·88	
**Merida**	0·5-1M		0·76	0·91			0·73	0·82			0·62	0·82	
**Veracruz**	0·5-1M		0·71	0·93			0·38	0·89			0·38	0·90	
**Cancun**	0·5-1M		0·89	0·93			0·71	0·82			0·67	0·83	
**Tapachula**	<0·5M		0·79	0·95			0·33	0·88			0·33	0·89	
**Villahermosa**	<0·5M		0·95	0·95			0·75	0·86			0·85	0·88	
**Campeche**	<0·5M		0·71	0·94			0·5	0·85			0·31	0·83	
**Iguala**	<0·5M		0·63	0·98			0·67	0·93			0·42	0·92	
**Coatzacoalcos**	<0·5M		0·8	0·96			0·33	0·89			0·36	0·91	
**mean (sd)**			0·77	0·95			0·56	0·87			0·51	0·87	

*Separation of cities based on their population size: 0·5-1M are cities with population between 500,000 and 1,000,000 whereas <0·5M are cities with population below 500,000.

Table S4. Percentage of total population, area, number of census tracts and cases of each disease from each city that occurred inside hotspot areas.

		**% of totals for each city**							
**City**	**Strata***	**Population**	**area**	**Census tract**	**DEN**	**CHIK 2015**	**CHIK 2016**	**ZIK 2016**	**All viruses**
Acapulco	0·5-1M	31•4	16•1	19•0	40•4	34•5	44•8	37•3	38•6
Mérida	0·5-1M	31•8	22•8	18•9	41•2	35•5	25•5	33•1	35•9
Veracruz	0·5-1M	14•9	11•4	9•10	19•8	15•6	20•7	17•0	18•3
Cancún	0·5-1M	28•5	17•3	18•1	34•1	26•9	39•5	19•1	32•6
Tapachula	<0·5M	16•3	15•7	9•80	24•3	21•5	20•7	16•8	23•2
Villahermosa	<0·5M	27•0	18•1	17•9	41•1	63•2	33•3	37•9	39•5
Campeche	<0·5M	24•4	23•2	18•1	32•5	35•1	32•4	27•8	30•5
Iguala	<0·5M	43•0	22•8	18•1	47•9	54•9	52•2	51•8	50•5
Coatzacoalcos	<0·5M	12•8	10•8	11•8	22•8	14•0	8•80	17•7	19•6
Mean (sd)	<0·5M	25•6 (9•7)	17•6 (4•7)	15•6 (4•1)	33•8(9•7)	33•5(16•7)	30•9 (13•5)	28•7(12•3)	32•1(10•5)

*Separation of cities based on their population size: 0.5-1M are cities with population between 500,000 and 1,000,000 whereas <0.5M are cities with population below 500,000.

Table S5. Spatial agreement in the Distribution of case counts of DENV, CHIKV and ZIKV in the nine cities of study. Cell values show the Kendall *W* (and p-value) for each disease pair combination. Non-significant (P>0.05) associations are shown in bold.

City	Strata*	DENV-CHIKV15	DENV-CHIKV16	DENV-ZIKV15	ZIKV16-CHIKV15	ZIKV16-CHIKV16	CHIKV15-CHIKV16
Acapulco	0·5-1M	0·781(<0·01)	0·746(<0·01)	0·886(<0·01)	0·813(<0·01)	0·795(<0·01)	0·731(<0·01)
Mérida	0·5-1M	0·773(<0·01)	0·757(<0·01)	0·869(<0·01)	0·757(<0·01)	0·782(<0·01)	0·673(<0·01)
Veracruz	0·5-1M	0·726(<0·01)	0·733(<0·01)	0·872(<0·01)	0·733(<0·01)	0·779(<0·01)	0·703(<0·01)
Cancún	0·5-1M	**0·549(0·11)**	**0·562(0·064)**	0·632(<0·01)	0·582(<0·01)	0·675(<0·01)	0·609(<0·01)
Tapachula	<0·5M	0·727(<0·01)	0·684(<0·01)	0·847(<0·01)	0·706(<0·01)	0·665(<0·01)	0·634(0·02)
Villahermosa	<0·5M	0·606(0·07)	0·77(<0·01)	0·792(<0·01)	**0·586(0·11)**	0·795(<0·01)	**0·555(0·207)**
Campeche	<0·5M	0·724(<0·01)	0·682(<0·01)	0·857(<0·01)	0·696(<0·01)	0·776(<0·01)	0·652(0·02)
Iguala	<0·5M	0·772(<0·01)	0·809(<0·01)	0·923(<0·01)	0·771(<0·01)	0·802(<0·01)	0·742(<0·01)
Coatzacoalcos	<0·5M	0·651(0·03)	0·717(<0·01)	0·893(<0·01)	0·677(0·02)	0·737(<0·01)	**0·578(0·16)**

*Separation of cities based on their population size: 0·5-1M are cities with population between 500,000 and 1,000,000 whereas <0·5M are cities with population below 500,000.

Figure S1. Cumulative yearly rainfall by year and city for the period 2000-2015 (dots) inferred using a LOESS smoothing (blue line, mean value, gray bands, 95% CI).

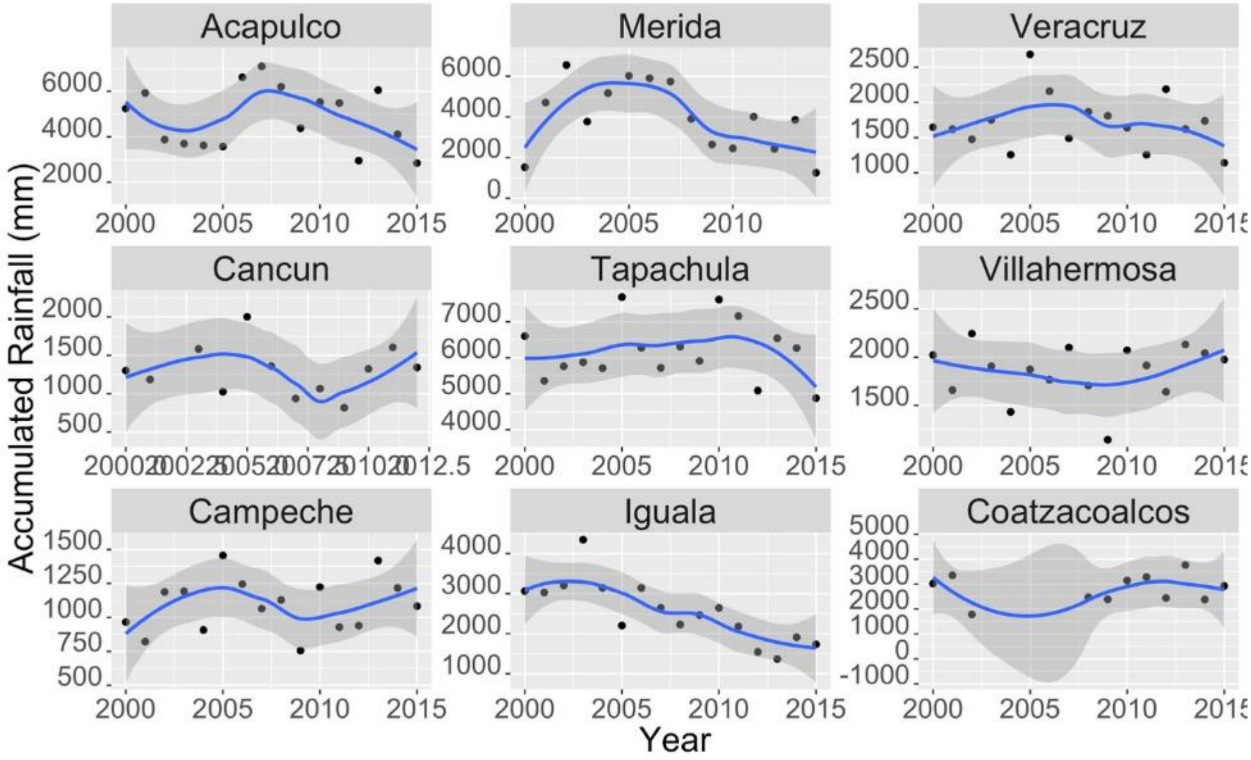

Figure S2. Average temperature the nine studied localities by epidemiological week. The maximum and minimum temperatures are represented by gray and black lines, respectively.

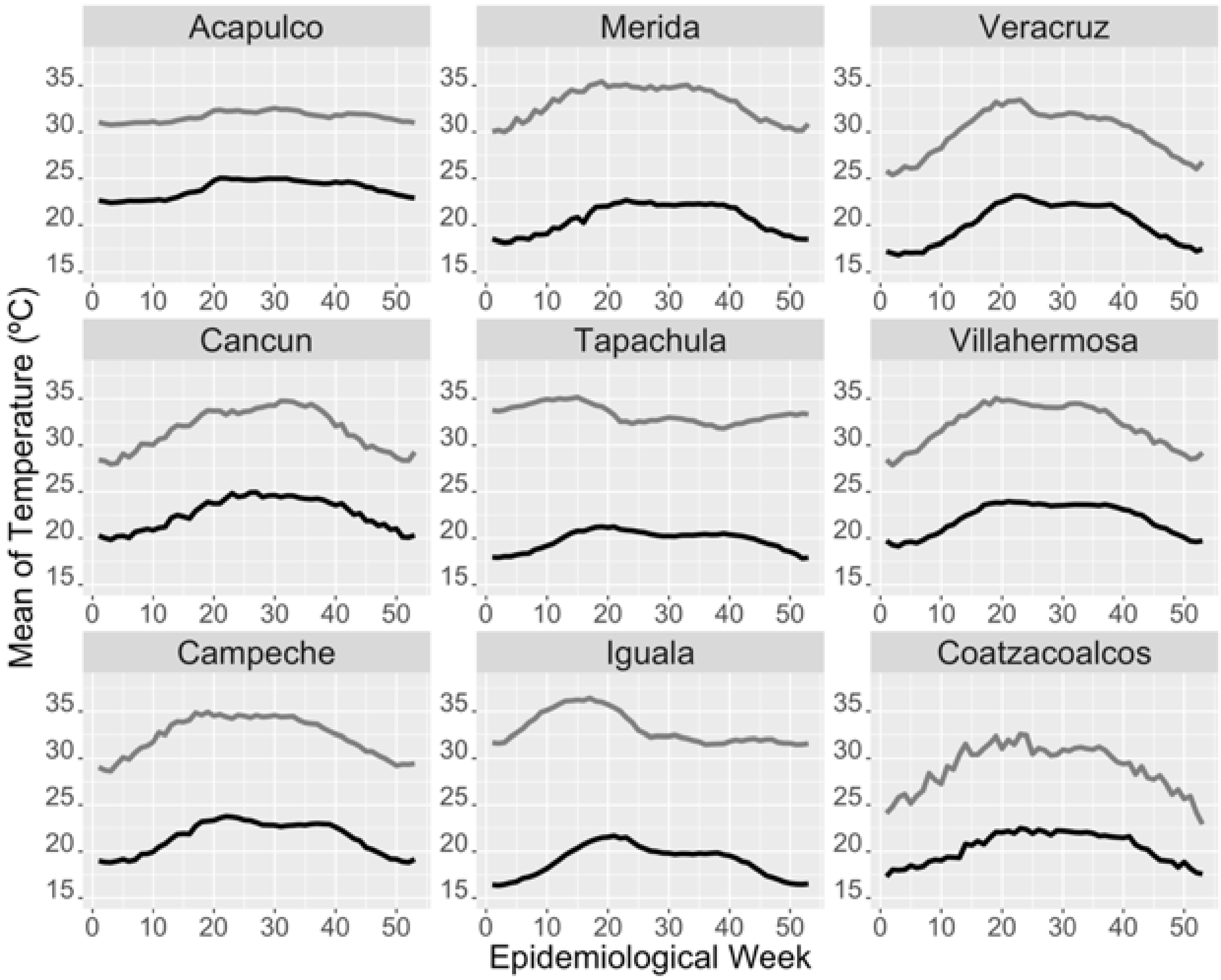

Figure S3. Seasonal dengue transmission by epidemiological week, year and locality. The number of cases is on y-axis and epidemiological weeks on the x-axis. The colors of lines indicate the year (2008-2016) and each box the studied localities. Vertical gray dotted lines indicate the peak of transmission that coincides with the rainy season.

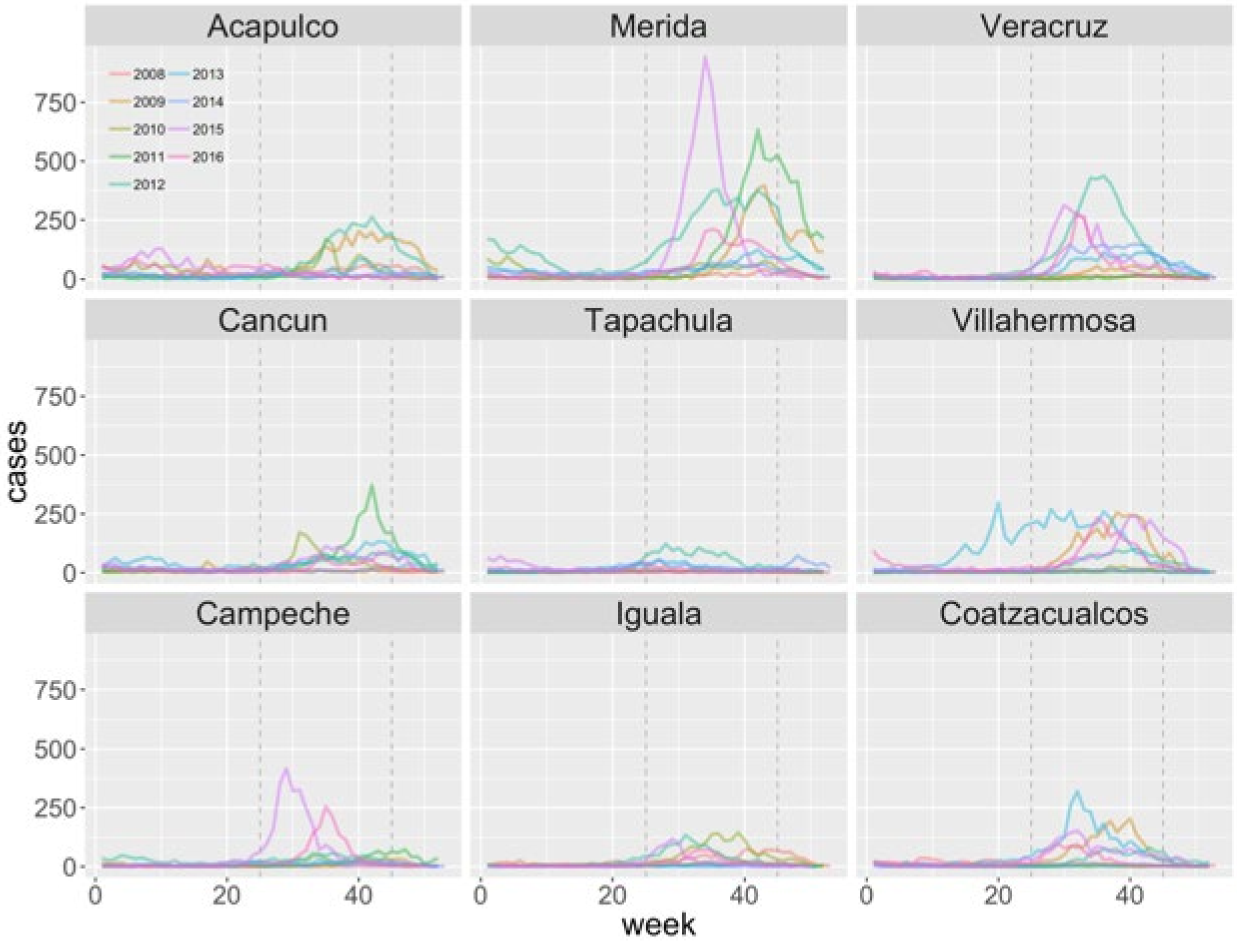

Figure S4. Seasonal chikungunya transmission by epidemiological week, year and locality. The number of cases is on y-axis and epidemiological weeks on the x-axis. The colors lines indicate the years (2015-2016) and each box the studied localities. Vertical gray dotted lines indicate the peak of transmission that coincides with the rainy season.

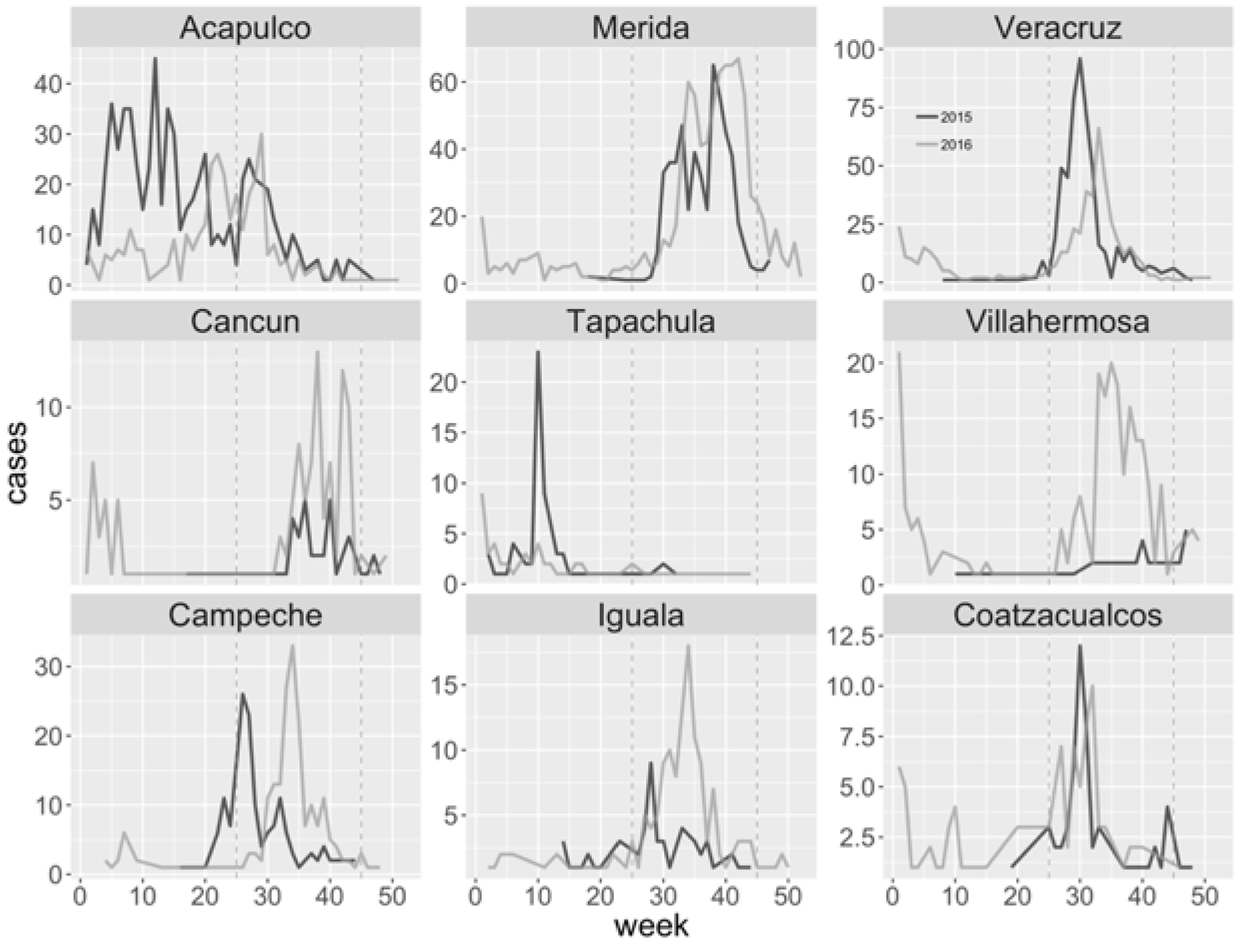

Figure S5. Seasonal Zika transmission by epidemiological week, year and city. The number of cases is on y-axis and epidemiological weeks on the x-axis. Vertical gray dotted lines indicate the peak of transmission that coincides with the rainy season.

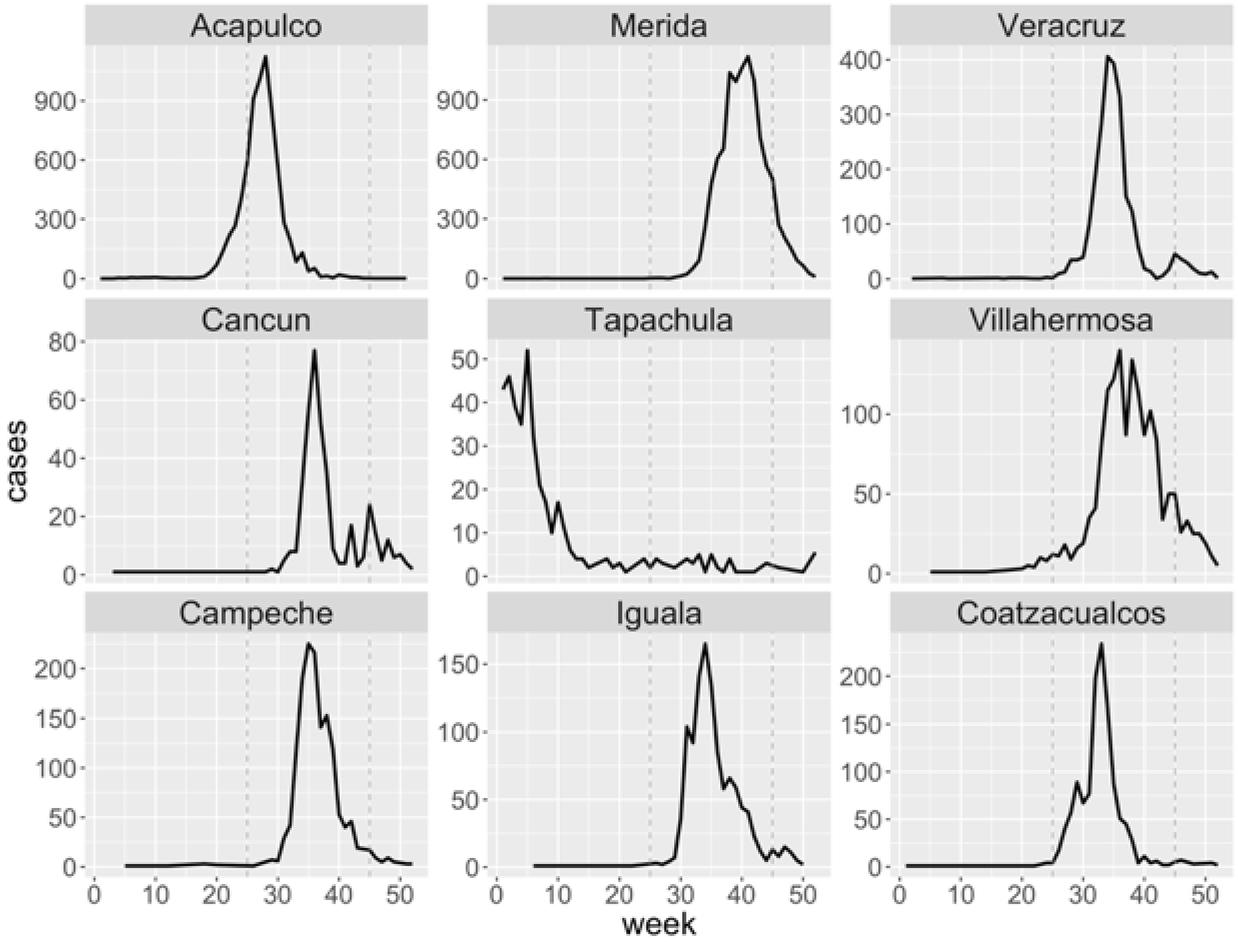

Figure S6. Relative distribution of DEN serotypes by city and year.

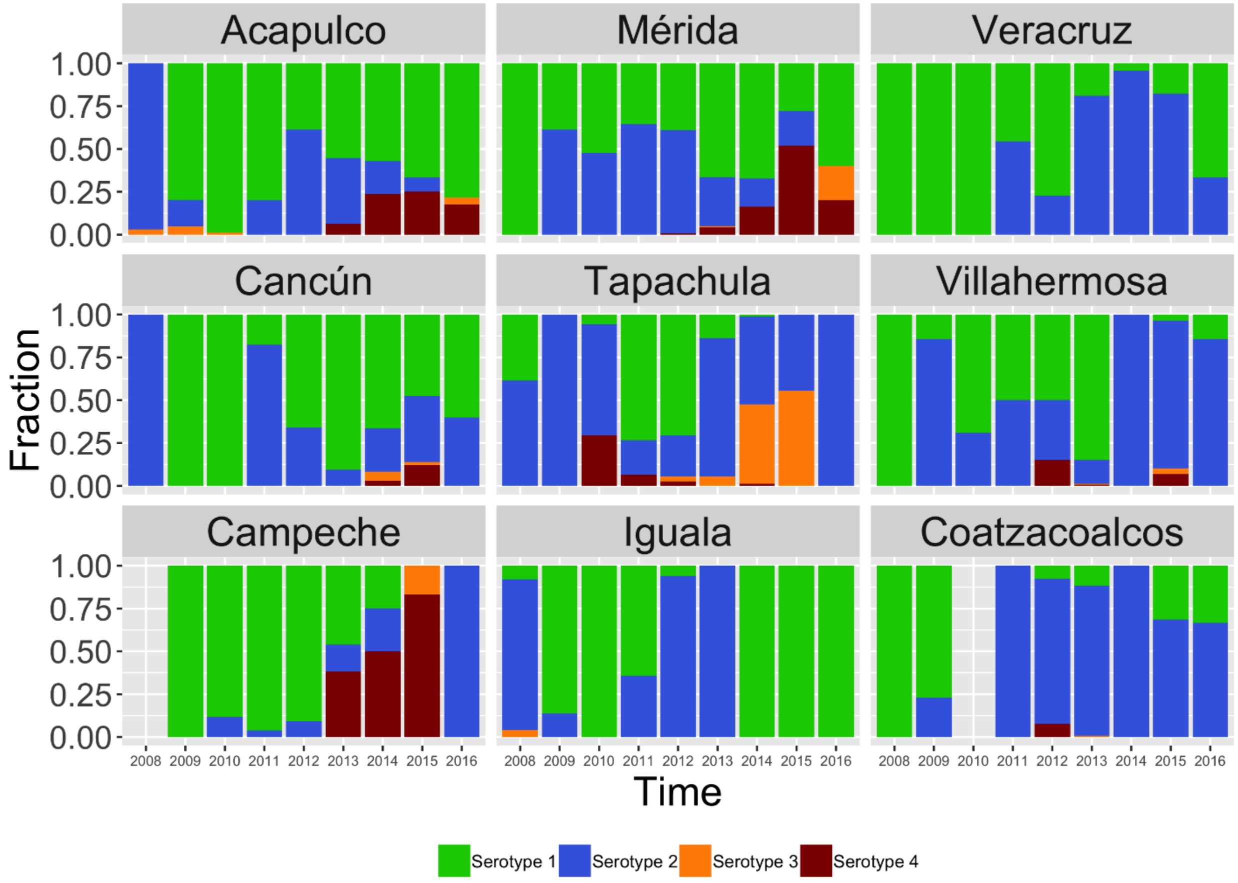

Figure S7. Schema of the data management and analysis plan.

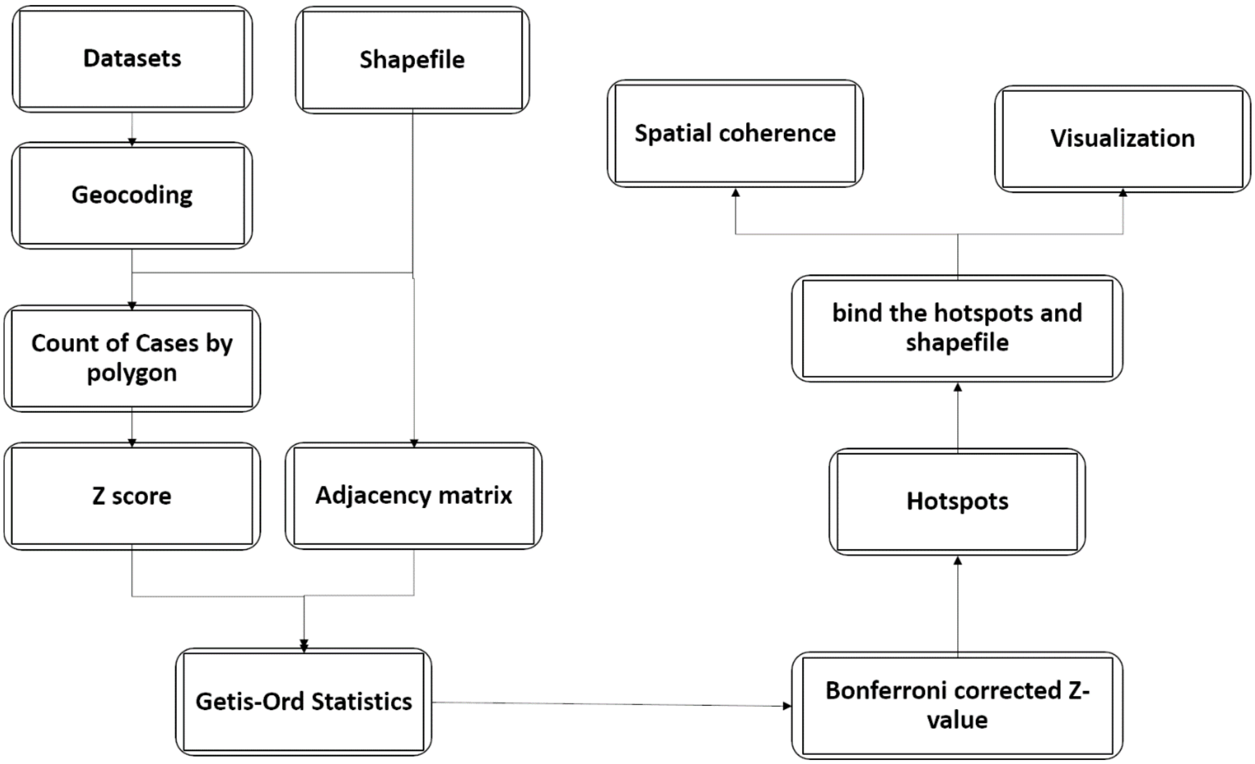

Figure S8. Z-score of dengue 2008 cases by locality and census tract.

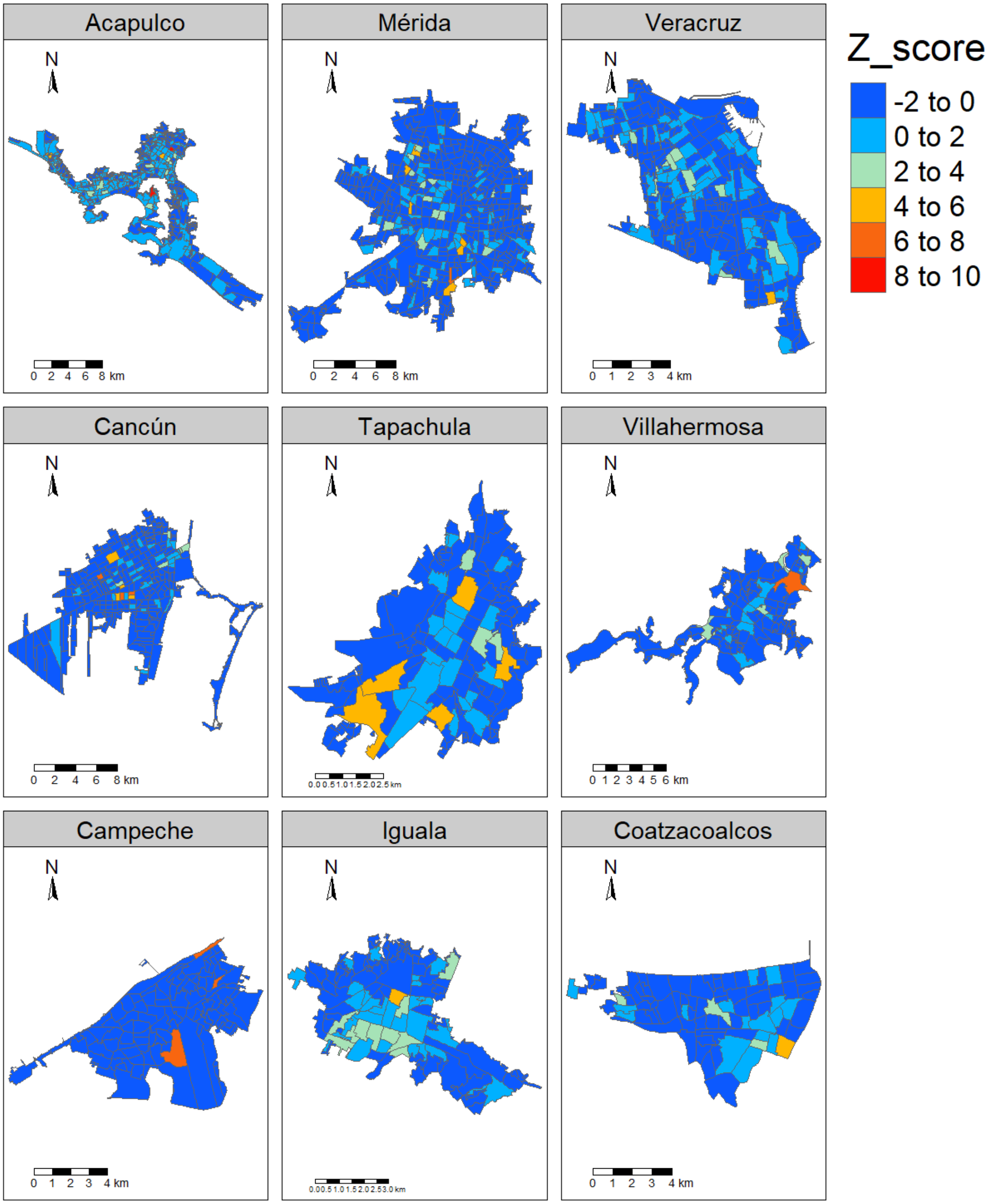

Figure S9. Z-score of dengue 2009 cases by locality and census tract.

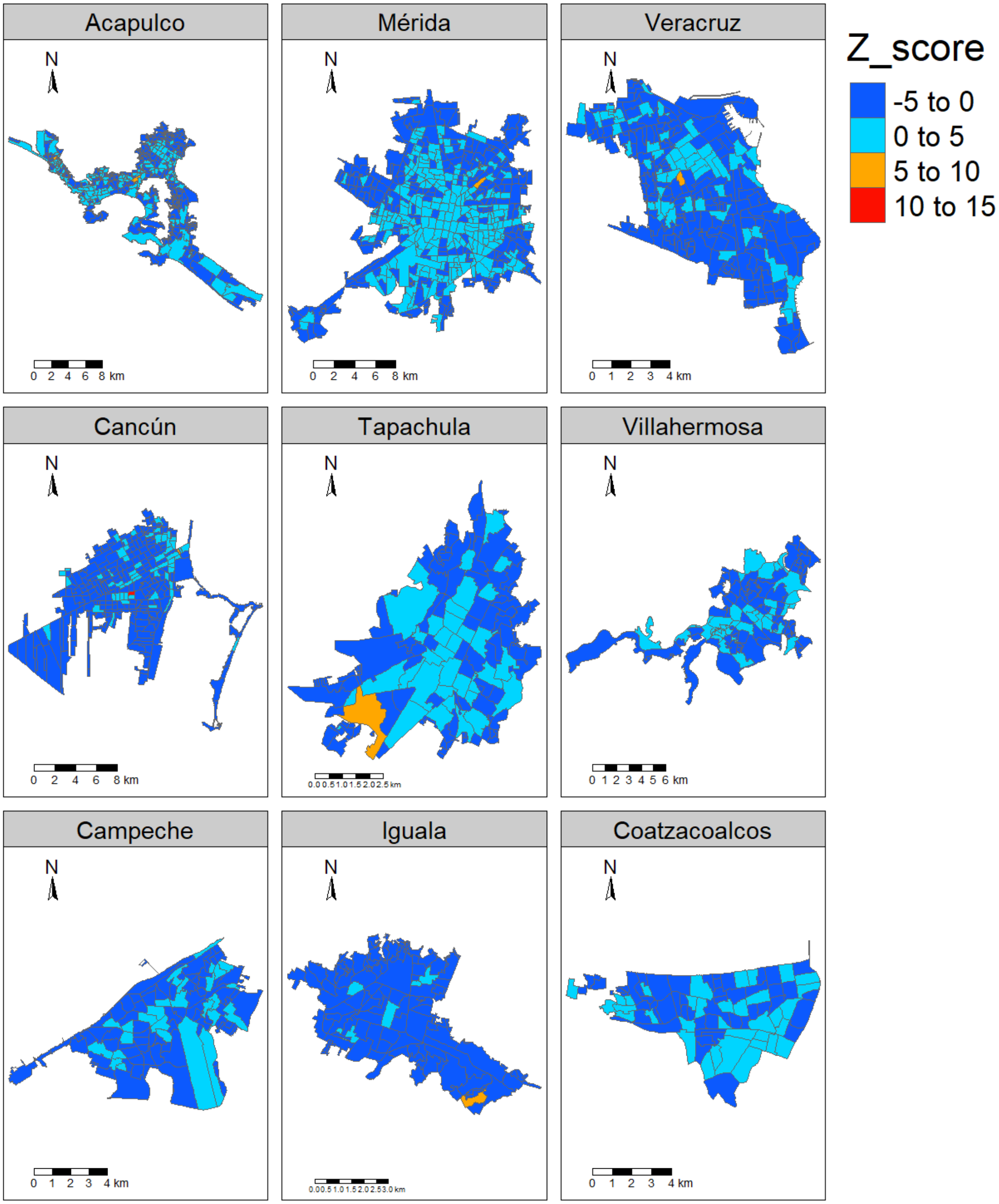

Figure S10. Z-score of dengue 2010 cases by locality and census tract.

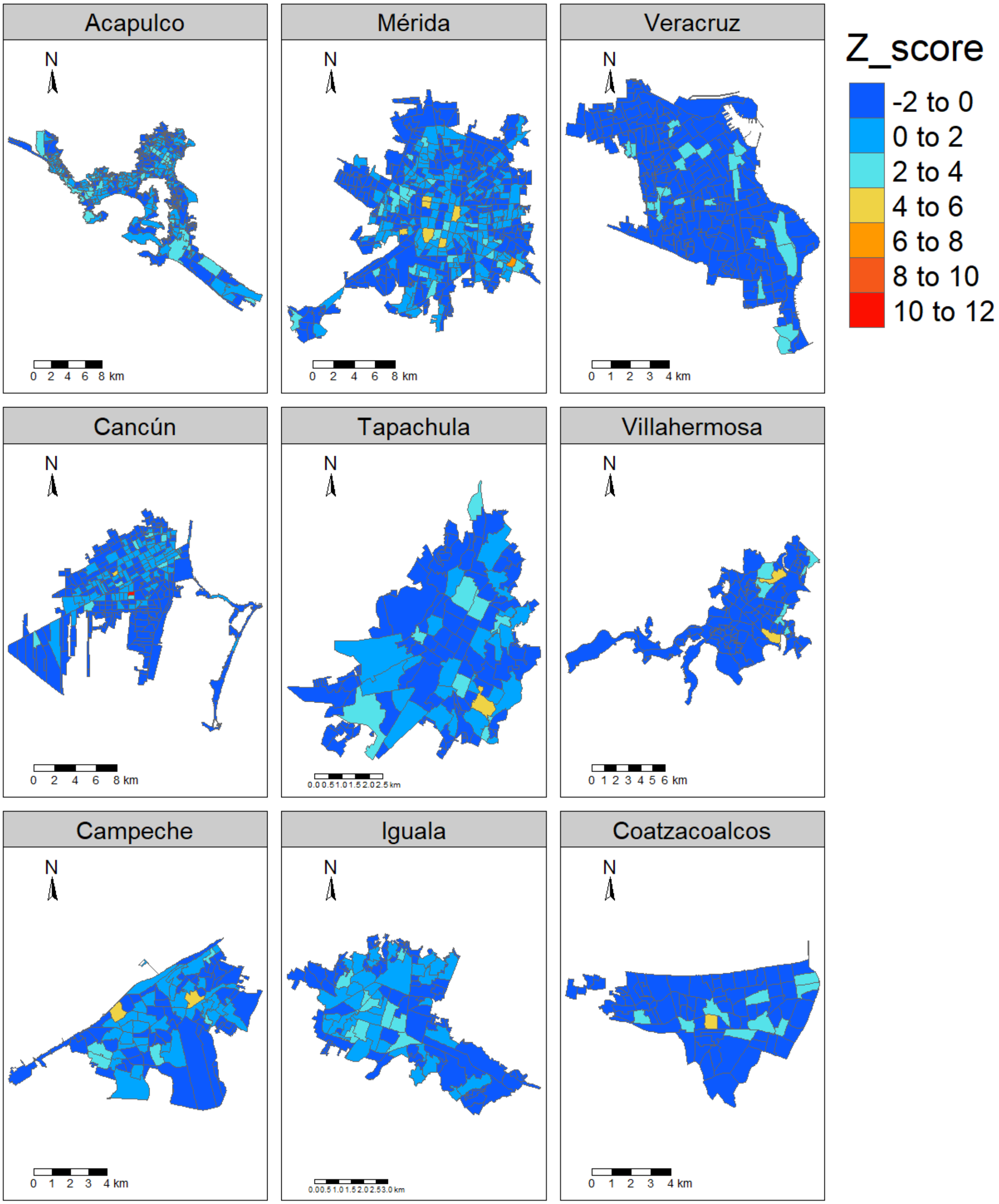

Figure S11. Z-score of dengue 2011 cases by locality and census tract.

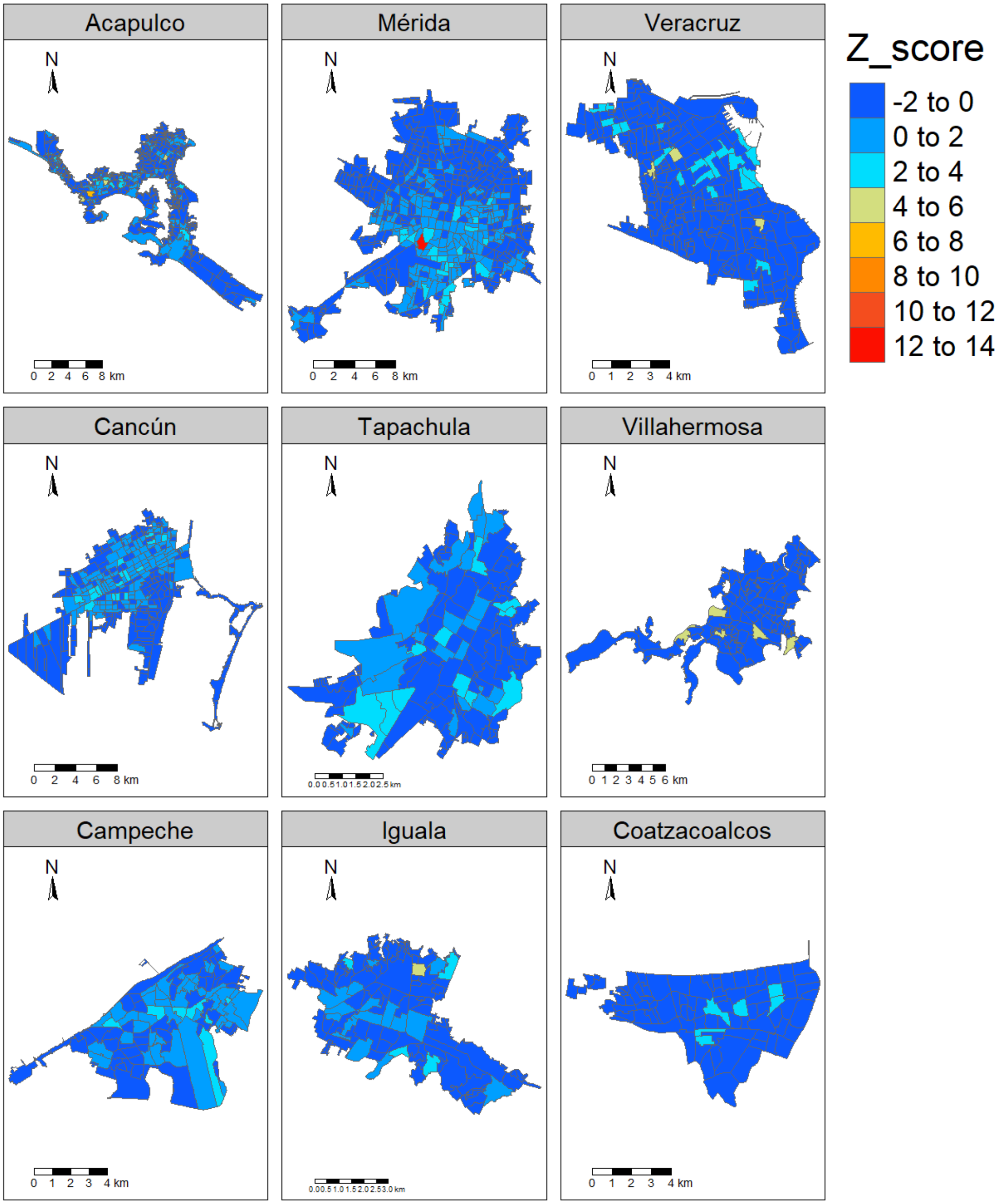

Figure S12. *Z*-score of dengue 2012 cases by locality and census tract.

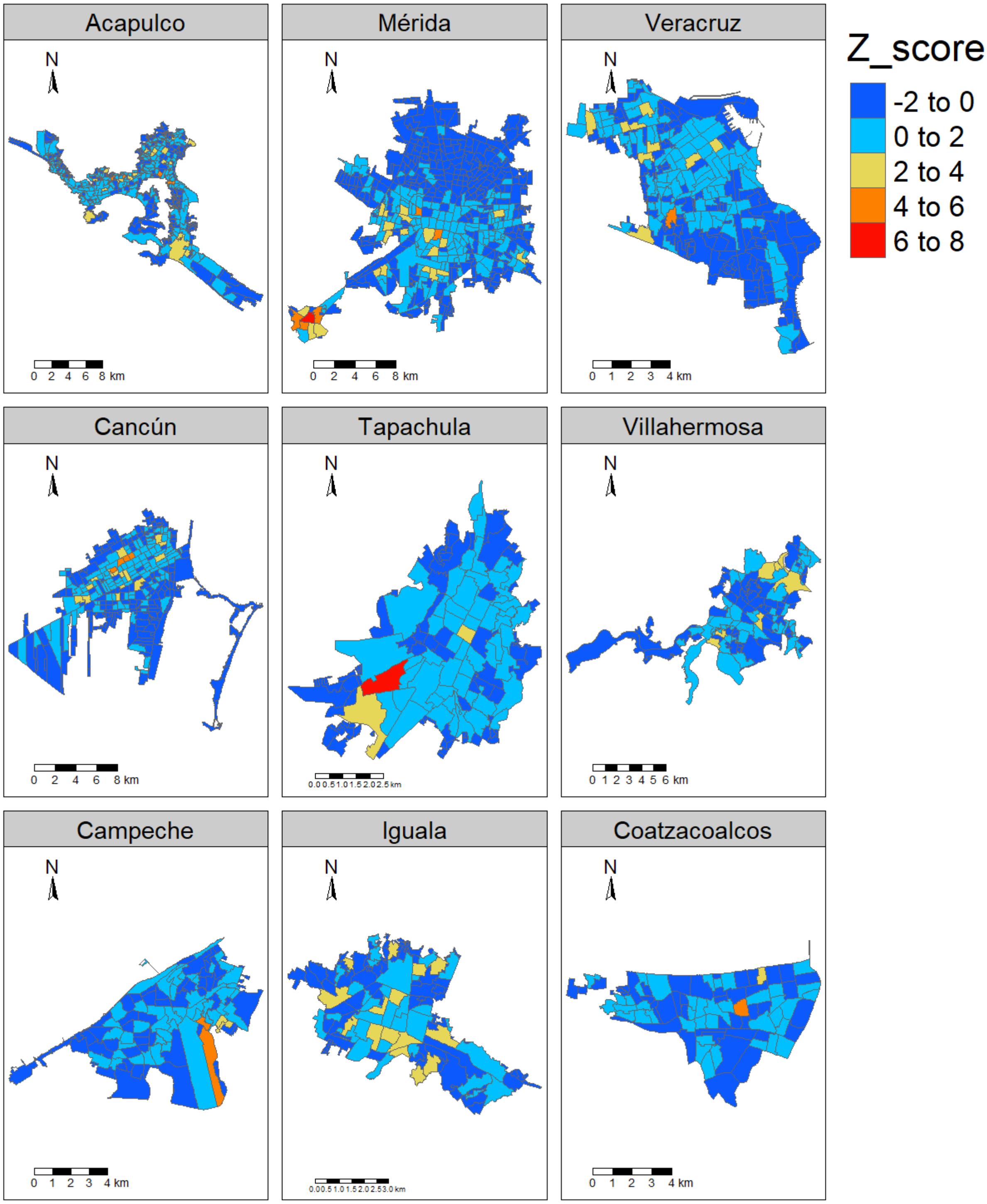

Figure S13. Z-score of dengue 2013 cases by locality and census tract.

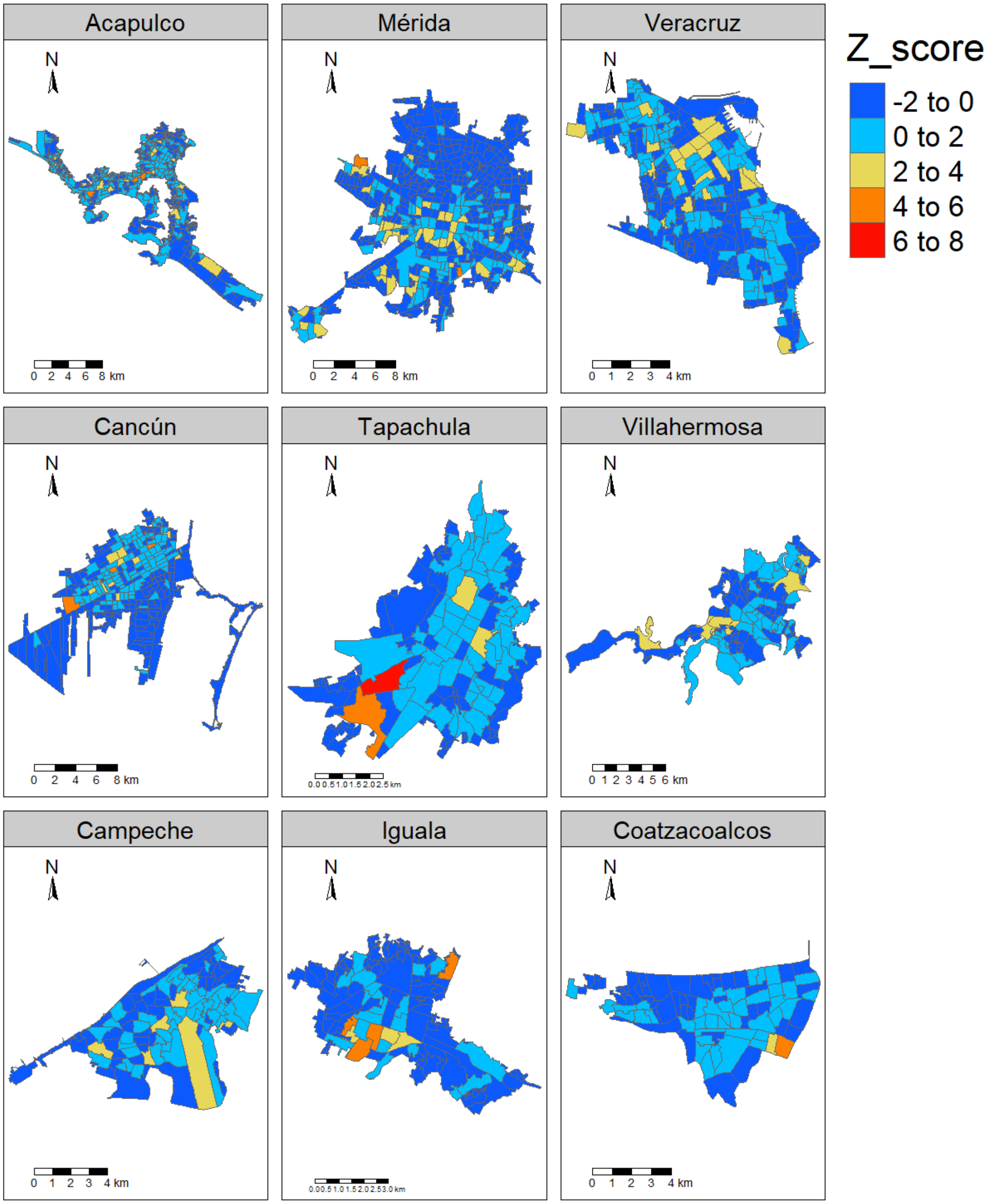

Figure S14. Z-score of dengue 2014 cases by locality and census tract.

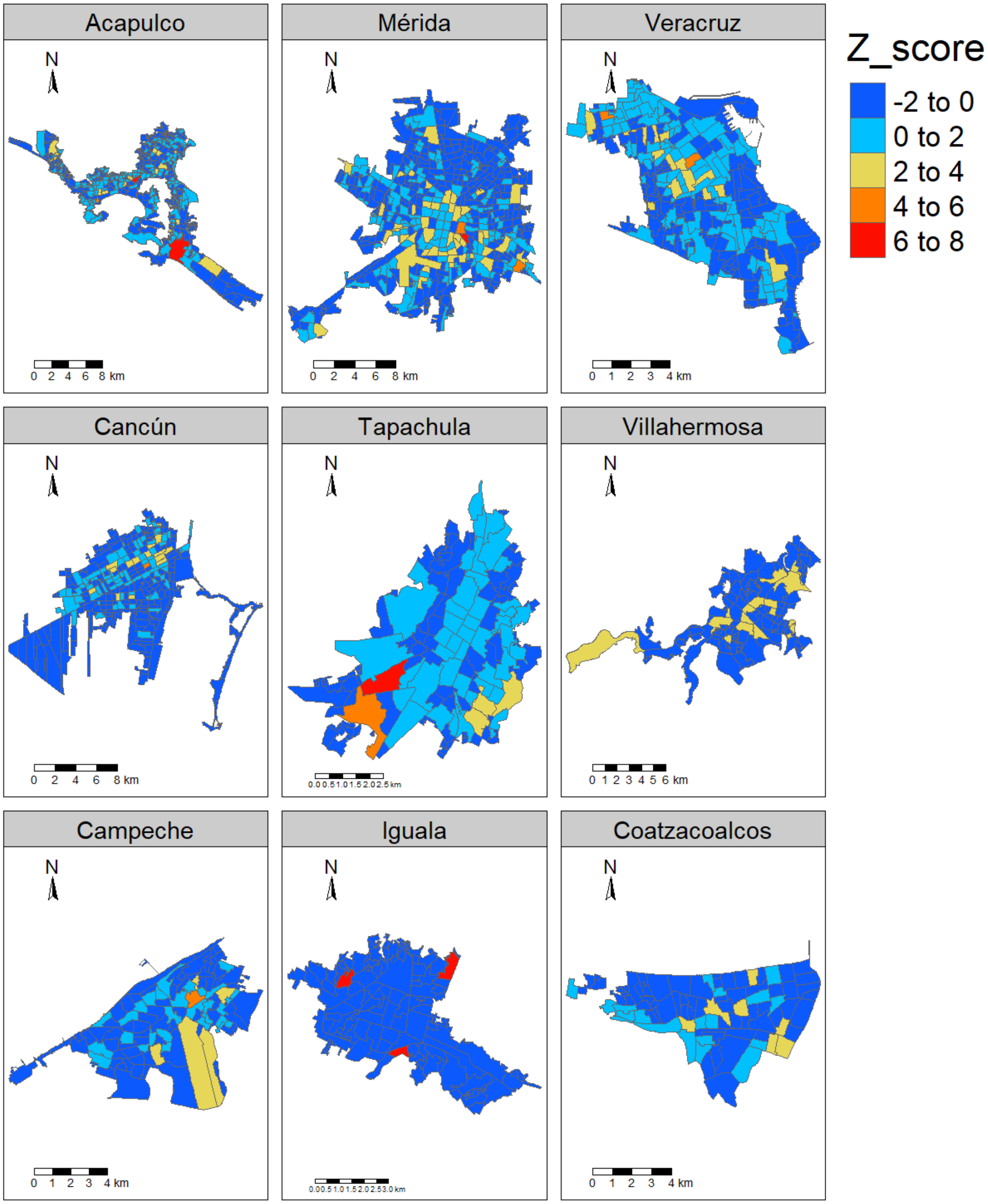

Figure S15. Z-score of dengue 2015 cases by locality and census tract.

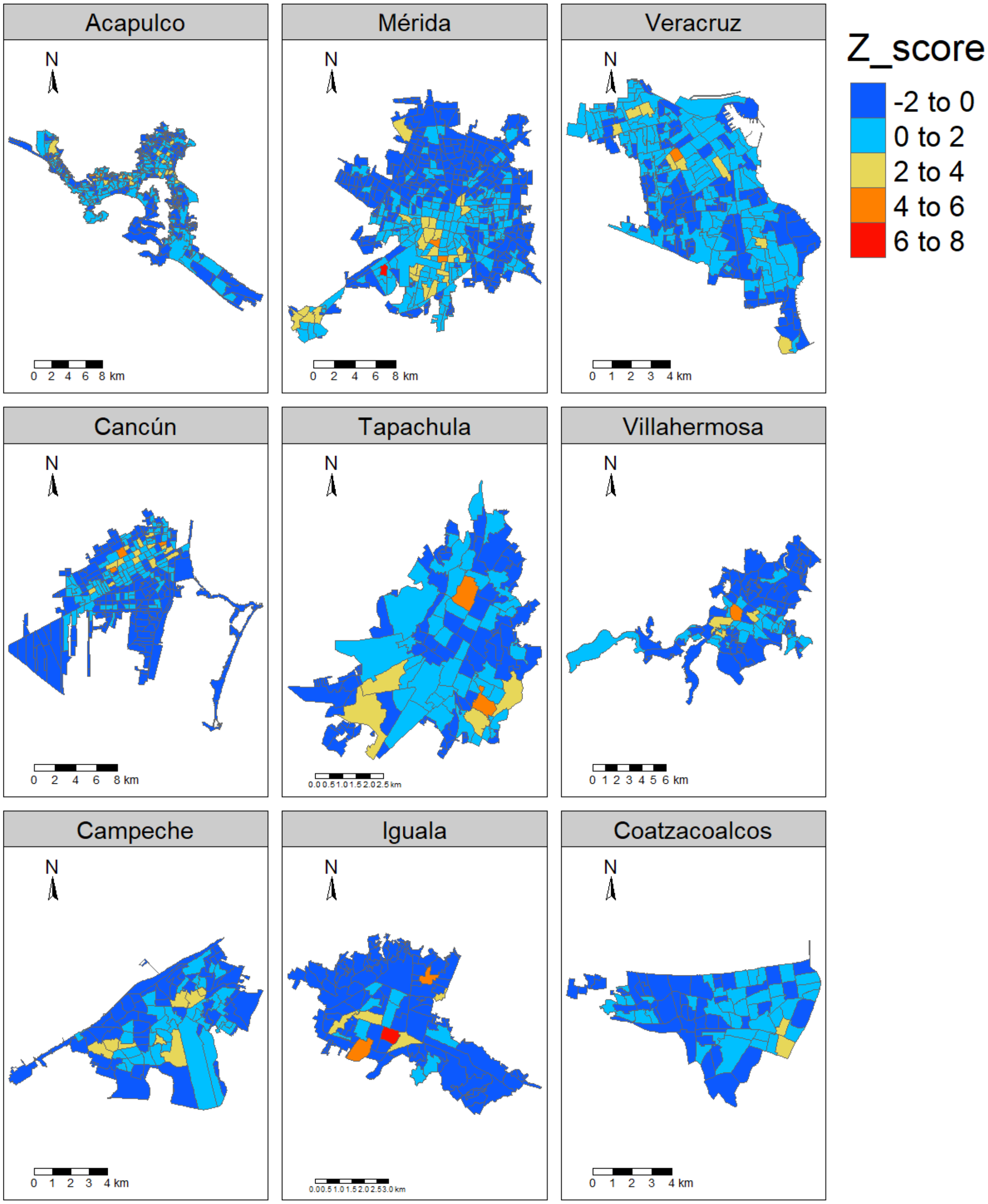

Figure S16. Z-score of dengue 2016 cases by locality and census tract.

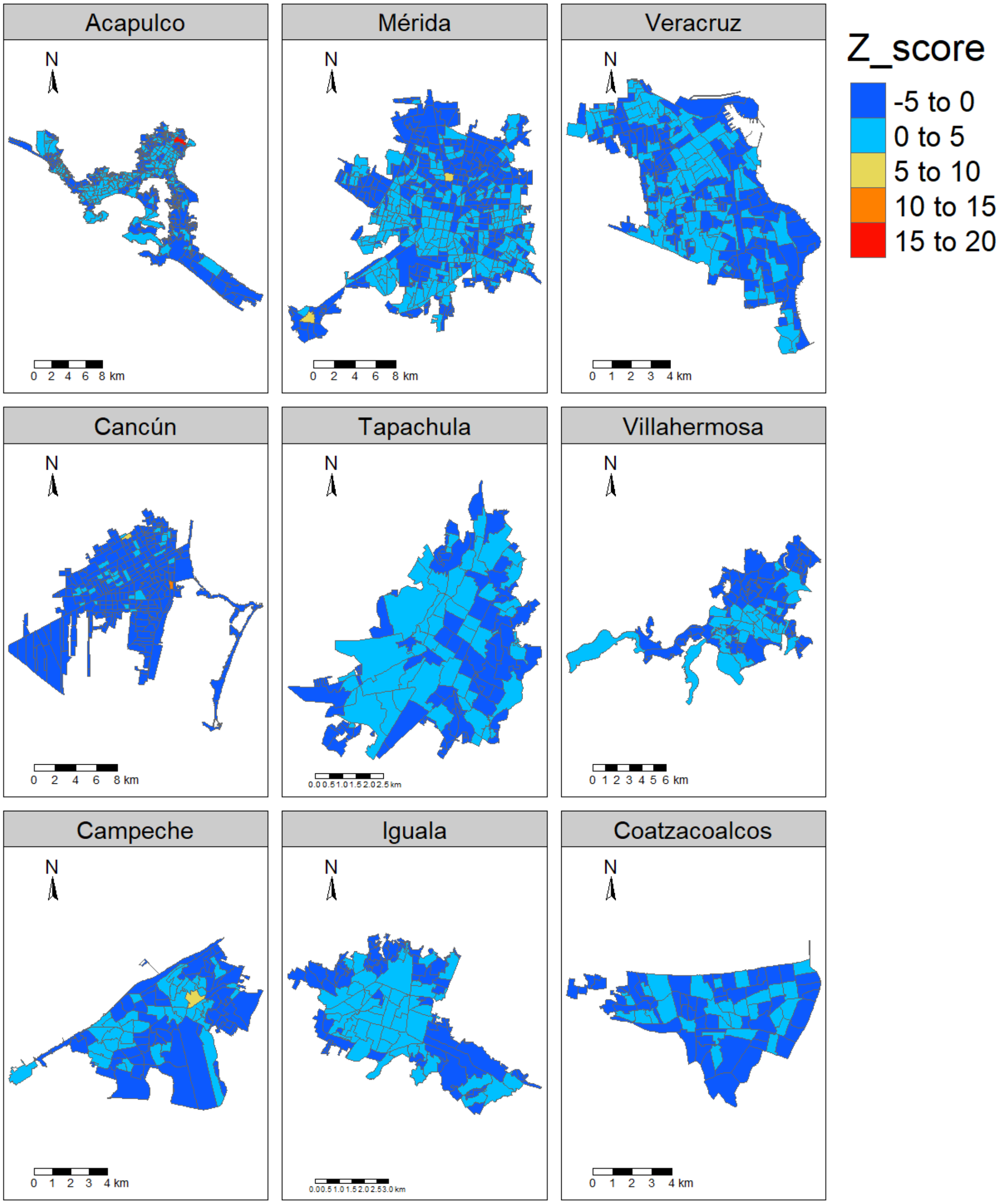

Figure S17. Z-score of chikungunya 2015 cases by locality and census tract.

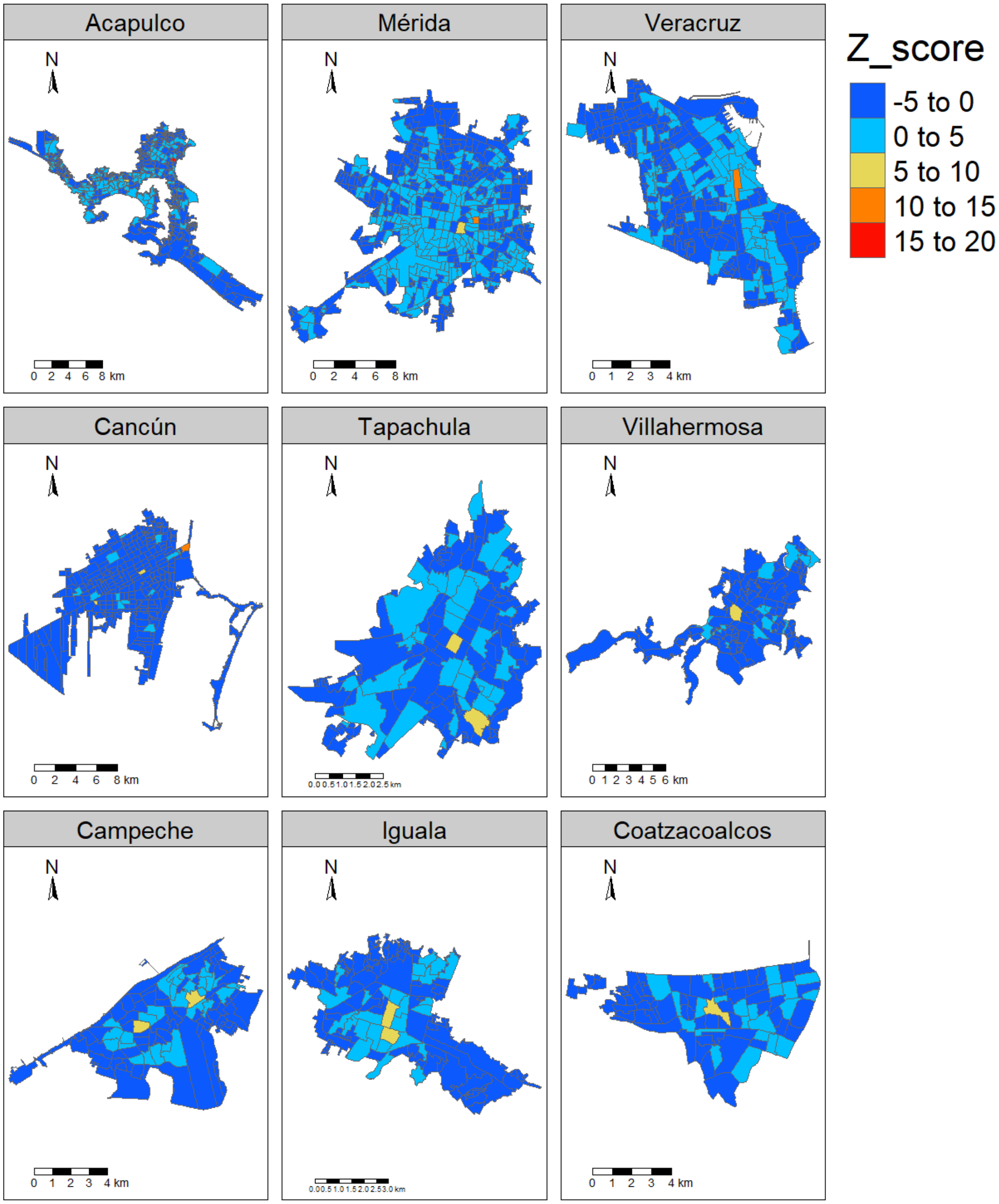

Figure S18. Z-score of chikungunya 2016 cases by locality and census tract.

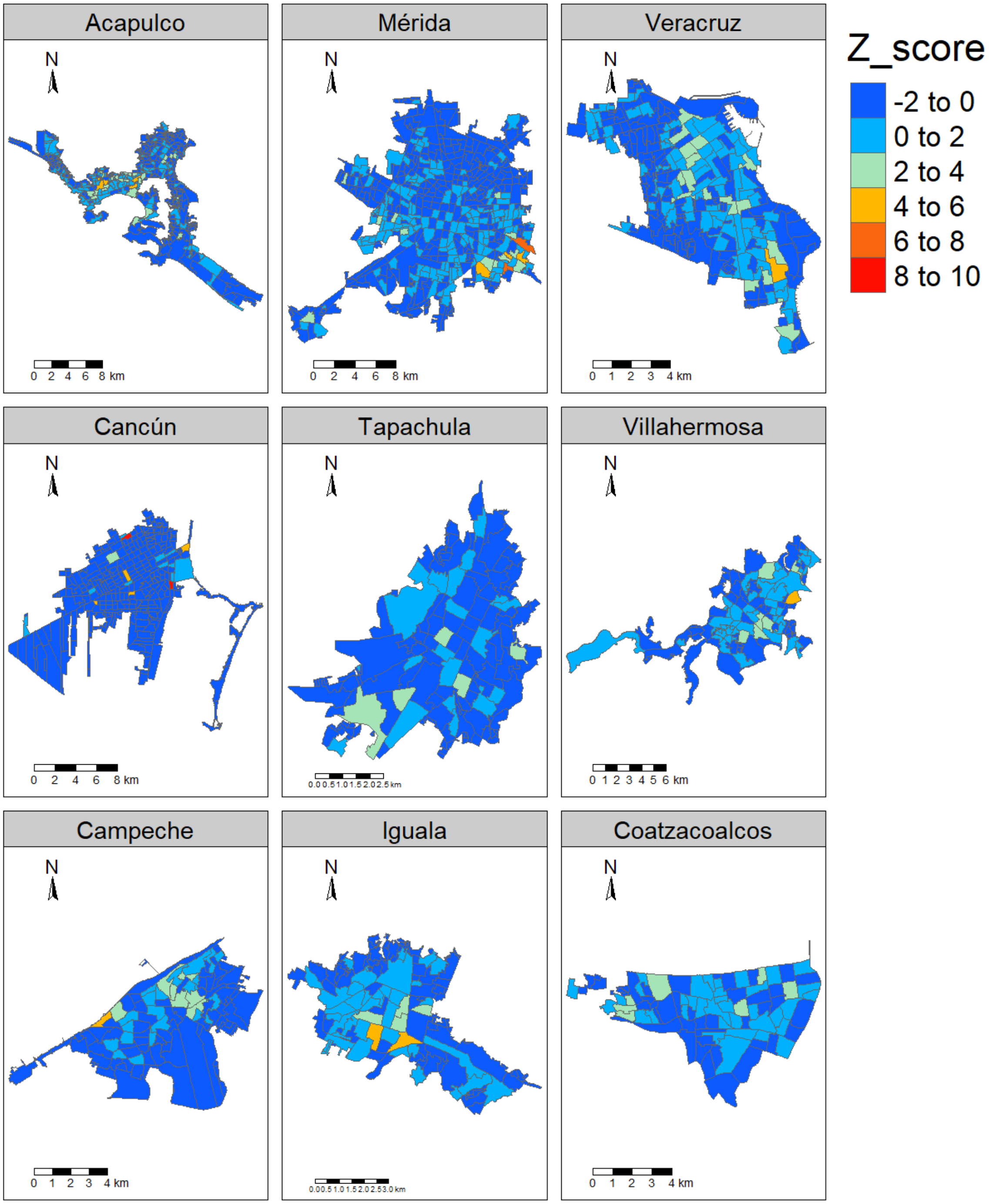

Figure S19. Z-score of zika 2016 cases by locality and census tract.

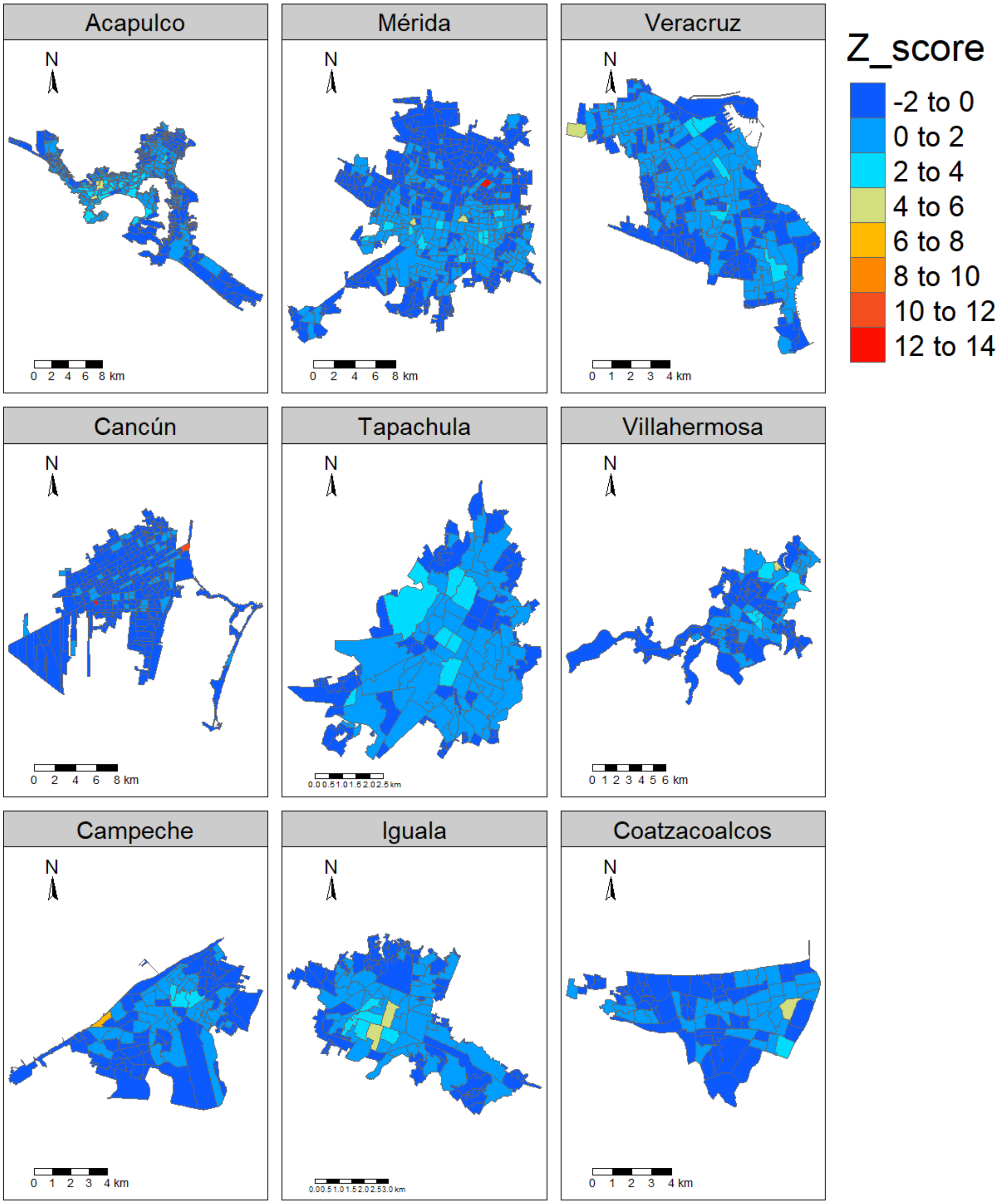

Figure S20. *Z*-score for dengue cases by census unit, split by city and year (2017-2020). This dataset was used to validate hotspot locations found during 2008-2016.

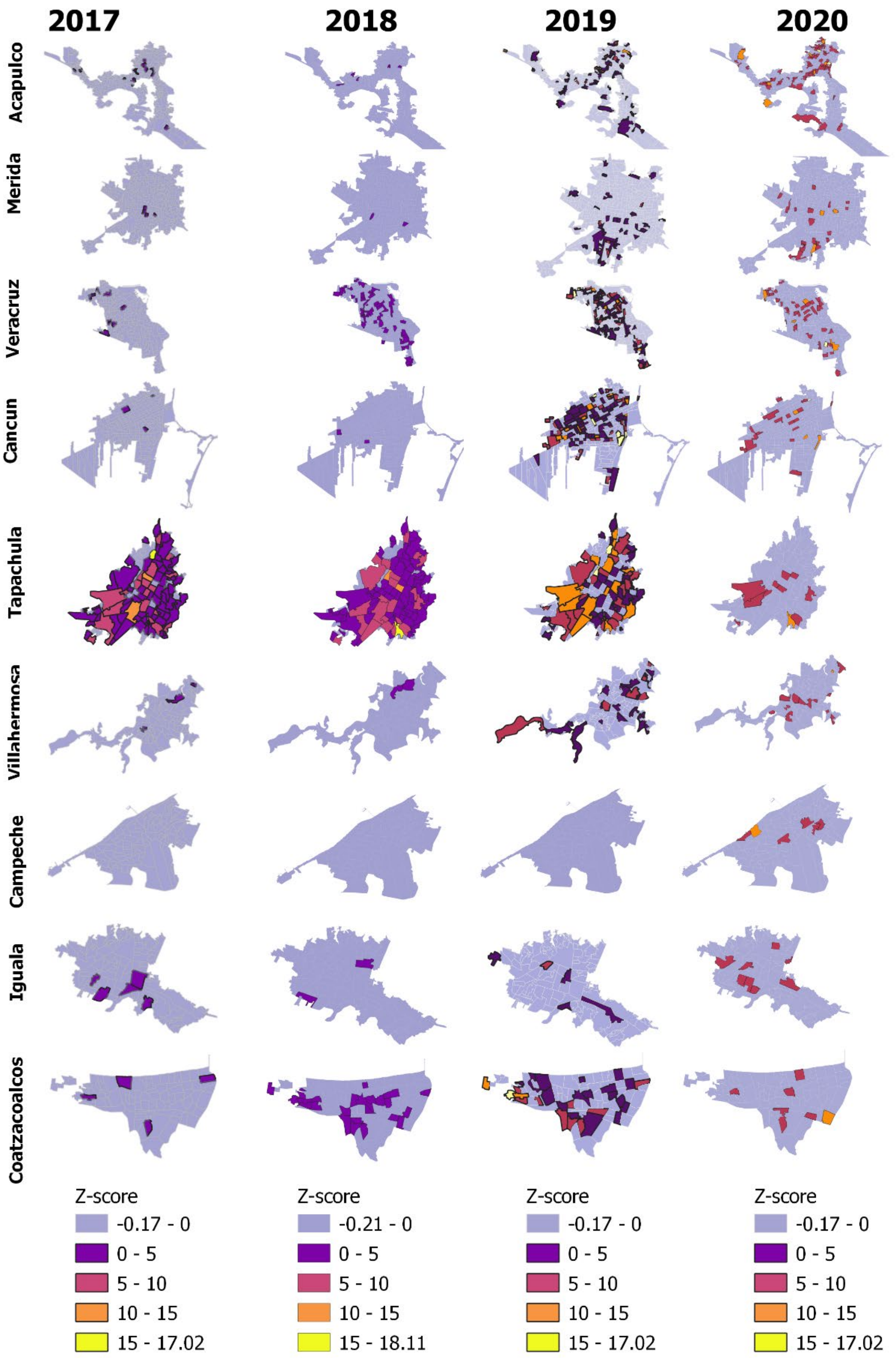

Supplement: Supplementary appendix 2 [file mmc2.pdf]
